# TMEM106A as a Macrophage‐Associated Biomarker of Prognosis in IDH‐Wildtype Glioma: Integrative Multi‐Omics and Spatial Analyses

**DOI:** 10.1002/cam4.71454

**Published:** 2025-12-07

**Authors:** Wen‐Shin Song, Pei‐Chi Chang, Dueng‐Yuan Hueng, Yao‐Feng Li

**Affiliations:** ^1^ Division of Neurosurgery, Department of Surgery Cheng‐Hsin General Hospital Taipei Taiwan; ^2^ Department of Neurological Surgery, Tri‐Service General Hospital National Defense Medical University Taipei Taiwan; ^3^ Graduate Institute of Life Sciences National Defense Medical University Taipei Taiwan; ^4^ Graduate Institute of Pathology and Parasitology National Defense Medical University Taipei ROC Taiwan; ^5^ Department of Pathology, Tri‐Service General Hospital National Defense Medical University Taipei Taiwan

**Keywords:** CGGA, glioma, Ivy dataset, pRRophetic, single‐cell sequencing, spatial‐transcriptomes, TCGA, TMEM106A

## Abstract

**Introduction:**

Gliomas remain aggressive despite current therapies, highlighting the urgent need for new biomarkers and targets. Transmembrane protein 106A (TMEM106A), implicated as a tumor suppressor in various cancers, has an unclear role in gliomas. We hypothesized that TMEM106A expression associates with tumor aggressiveness and may serve as a prognostic, microenvironmental biomarker.

**Methods:**

We integrated TCGA and CGGA bulk RNA‐seq, single‐cell (GSE131928, GSE89567), spatial (Ivy Atlas, Visium), and immunohistochemistry (*n* = 79) to evaluate TMEM106A. Differential expression used limma. Survival used Kaplan–Meier and multivariable Cox models. Immune contexture used a 12‐cell‐state deconvolution and CIBERSORT. GSEA assessed hallmark pathways. Drug sensitivity was inferred using pRRophetic. Immunotherapy modeling combined TCGA expression with TCIA immunophenoscore and PD‐L1.

**Results:**

(1) TMEM106A mRNA is significantly upregulated in high‐grade gliomas compared to lower‐grade gliomas and normal brain. (2) In IDH‐wildtype tumors, differential analyses highlight roles of TMEM106A and TMEM106C; high expression links to poorer prognosis. (3) TMEM106A is an independent prognostic factor associated with aggressive behavior, especially in IDH‐wildtype astrocytomas. (4) Upregulation is confirmed by immunohistochemistry. (5) High TMEM106A associates with pro‐inflammatory signatures and higher inferred fractions of myeloid cells and granulocytes. (6) Single‐cell RNA‐seq shows enrichment in myeloid lineages, and (7) CIBERSORT shows modest positive correlations with polarized macrophage signatures. (8) Spatial transcriptomics shows higher TMEM106A in myeloid‐rich regions, consistent with a microenvironmental readout. (9) In IDH‐wildtype tumors, pRRophetic predicts lower IC50 for multiple targeted agents in TMEM106A‐high tumors. (10) TMEM106A‐high IDH‐wildtype tumors show higher IPS only when PD‐1 is “on,” suggesting a context‐dependent, inflamed‐but‐suppressed state.

**Conclusion:**

TMEM106A independently predicts survival and correlates with myeloid‐enriched transcriptional states in gliomas. Given its high expression in myeloid lineages in single‐cell data, bulk upregulation is potentially driven by myeloid infiltration rather than tumor‐cell intrinsic mechanisms. All findings are correlative; prospective studies are needed before any clinical use is considered.

**Abbreviations**
10x Visium: 10x Genomics Visium spatial transcriptomics
AP‐MS: affinity‐purification mass spectrometry
BH: Benjamini–Hochberg (multiple‐testing correction)
CGGA: Chinese Glioma Genome Atlas
CGP2016: Cancer Genome Project 2016 (pRRophetic training set)
Chemo: chemotherapy
CIBERSORT: cell‐type identification by estimating relative subsets of RNA transcripts
FDR: false discovery rate
GEO: gene expression omnibus
GSEA: gene set enrichment analysis
IDHmu: IDH‐mutant astrocytomas
IDHwt: IDH‐wildtype astrocytomas
IPS: immunophenoscore
Karnofsky: Karnofsky Performance Score
LGG: lower‐grade glioma
Oligo: oligodendrogliomas
PD‐1/PD‐L1: programmed death‐1/Programmed death‐ligand 1 (gene proxy: CD274)
RPKM: reads per kilobase per million
RT: radiotherapy
TAM: tumor‐associated macrophage
TCGA: The Cancer Genome Atlas
TCIA: The Cancer Immunome Atlas
TMEM106A/B/C: transmembrane protein 106A/B/C
TMZ: temozolomide
TPM: Transcripts Per Million

## 1 Introduction

Gliomas are the most common malignant brain tumors in adults, and high‐grade gliomas (especially Glioblastoma, GBM) remain essentially incurable despite aggressive multimodal therapy [1]. Even with maximal surgical resection followed by chemoradiotherapy, patients have a poor prognosis, with a median survival of only 12–15 months for GBM [2]. This dismal outcome is attributed to the tumor's diffuse infiltrative growth, resistance to conventional therapies, and profound heterogeneity at the molecular and cellular levels [3]. In recent years, several molecular markers have been integrated into glioma classification and care. Notably, mutations in IDH1/2 and 1p/19q co‐deletion define distinct glioma subtypes, which exhibit better outcomes than IDH‐wildtype astrocytoma, also known as glioblastoma (GBM) [4]. Another important marker is *MGMT* promoter methylation, which predicts responsiveness to temozolomide chemotherapy [5]. Standard therapy for GBM—surgery followed by radiotherapy and temozolomide, and virtually all tumors recur [3]. Recurrent gliomas are often refractory to further treatment, leading to a uniformly fatal course. The challenges in treating gliomas stem from several factors: the infiltrative nature of glioma cells precludes complete surgical resection; the blood–brain barrier and limited tumor‐specific targets reduce the efficacy of systemic therapy; and the intrinsic genetic and phenotypic heterogeneity within gliomas fosters drug resistance and rapid adaptation. Despite decades of research, outcomes for patients with malignant glioma have improved only marginally. Furthermore, GBM's highly immunosuppressive microenvironment [6] contributes to therapeutic failure, as evidenced by the limited success of immunotherapies in this disease. Therefore, there is an urgent need for novel biomarkers and therapeutic targets to improve the prognostication and treatment of glioma patients [7, 8, 9]. Recently, through our bioinformatics analyses, we identified a potential biomarker, Transmembrane protein 106A (TMEM106A).

TMEM106A is an intriguing candidate biomarker that has not been studied in gliomas to date. *TMEM106A* is located on chromosome 17q21.31 and encodes a type II transmembrane protein predicted to localize to the cell membrane and mitochondria [10]. Its normal physiological function is not fully characterized, but it is conserved across mammals [10]. Nonetheless, limited prior evidence has linked the TMEM106 locus to glioma: an integrative TCGA multi‐omics classifier identified a DNA‐methylation block annotated to TMEM106A among features separating GBM from lower‐grade gliomas [11], and earlier GBM cancer‐stem‐cell datasets measured TMEM106A without reporting it as a significant hit [12]. In this study, we present a comprehensive analysis of TMEM106A in gliomas, integrating multi‐omics with single‐cell sequencing and spatial transcriptome datasets using advanced bioinformatics and immunohistochemistry. This design aligns with recent directions in pharmaceutical research, where AI‐enabled multi‐omics integration and digital pathology are accelerating biomarker discovery and therapy selection, and bibliometric analyses forecast sustained growth of such approaches [13, 14]. Through this multidisciplinary approach, we aim to determine whether TMEM106A can serve as a novel prognostic indicator and a potential candidate for glioma treatment strategies.

## 2 Materials and Methods

### 2.1 Data Collection for Bioinformatics Analyses

#### 2.1.1 Primary Bioinformatics Cohort (TCGA)

Transcriptomic (mRNA) profiles and associated clinical characteristics (gender, age, histology, tumor grade, overall survival, and vital status) were retrieved from The Cancer Genome Atlas (TCGA; https://portal.gdc.cancer.gov/). An initial set of 690 glioma cases was included under a 2007 classification framework [15]; these were subsequently reassigned according to the 2021 WHO classification system into three principal groups: IDH‐wildtype astrocytoma, IDH‐mutant astrocytoma, and oligodendroglioma [4].

#### 2.1.2 Tumor Heterogeneity and Immune Cell Analyses (TCGA and CGGA)

For deeper insights into tumor heterogeneity and immune infiltration, data from both the TCGA and Chinese Glioma Genome Atlas (CGGA; www.cgga.org.cn/, accessed December 2024) were analyzed.

#### 2.1.3 Ivy Glioblastoma Atlas

The Ivy Glioblastoma Atlas Project (https://glioblastoma.alleninstitute.org/) was next examined. This dataset comprises 122 samples collected from 10 patients across 5 heterogeneous tumor regions (leading edge, infiltrating border, cellular tumor, microvascular proliferation, and pseudopalisading necrosis) to investigate the spatial patterns of TMEM106A expression.

#### 2.1.4 Single‐Cell Sequencing (GEO)

Lastly, two single‐cell sequencing datasets from the Gene Expression Omnibus (GEO) were employed: one featuring IDH‐wildtype gliomas (GSE131928; https://www.ncbi.nlm.nih.gov/geo/query/acc.cgi?acc=GSE131928, accessed December 2024) and another covering IDH mutant gliomas (GSE89567; https://www.ncbi.nlm.nih.gov/geo/query/acc.cgi?acc=GSE89567, accessed December 2024).

#### 2.1.5 Figure–Data Source Map

**FIGURE 1:**
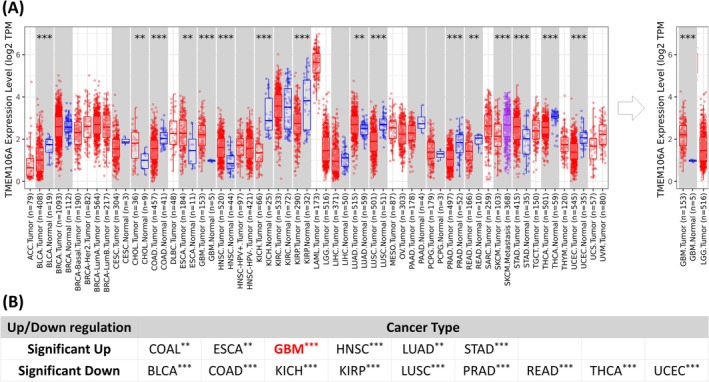
Pan‐cancer analysis of TMEM106A mRNA expression in TCGA. (A) Box plots compare tumor (red) and normal (blue) tissues for each cancer type, illustrating the 25th–75th percentiles, with medians indicated by horizontal lines and dots showing data spread. Asterisks mark significance levels (*p < 0.05, **p < 0.01, ***p < 0.001). In the extracted panel, the TMEM106A overexpression is especially notable in GBM. (B) The table summarizes these expression trends, categorizing each cancer type by the direction and significance of expression.

**FIGURE 2:**
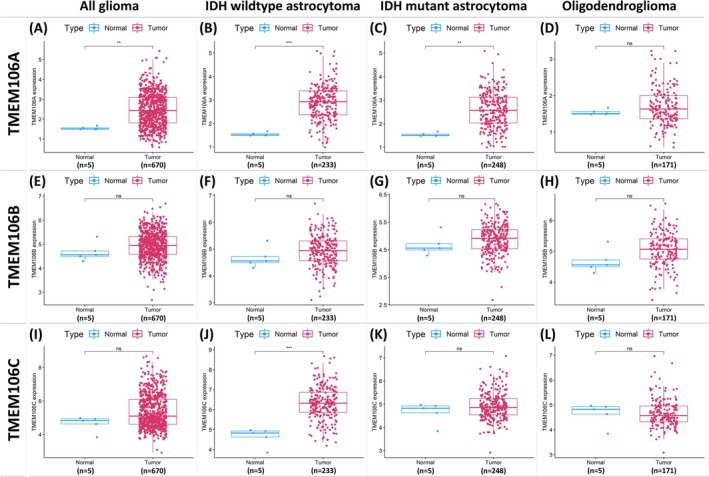
The mRNA differential analyses between normal and tumor of the TMEM106 family. (A–D) TMEM106A, (E–H) TMEM106B, and (I–L) TMEM106C in gliomas exhibit significant differences, as identified in the TMEM106A (A, B) and TMEM106C (J) groups (indicated by a red square), highlighting their potential roles. Asterisks mark significance levels (*p < 0.05, **p < 0.01, ***p < 0.001).

**FIGURE 3:**
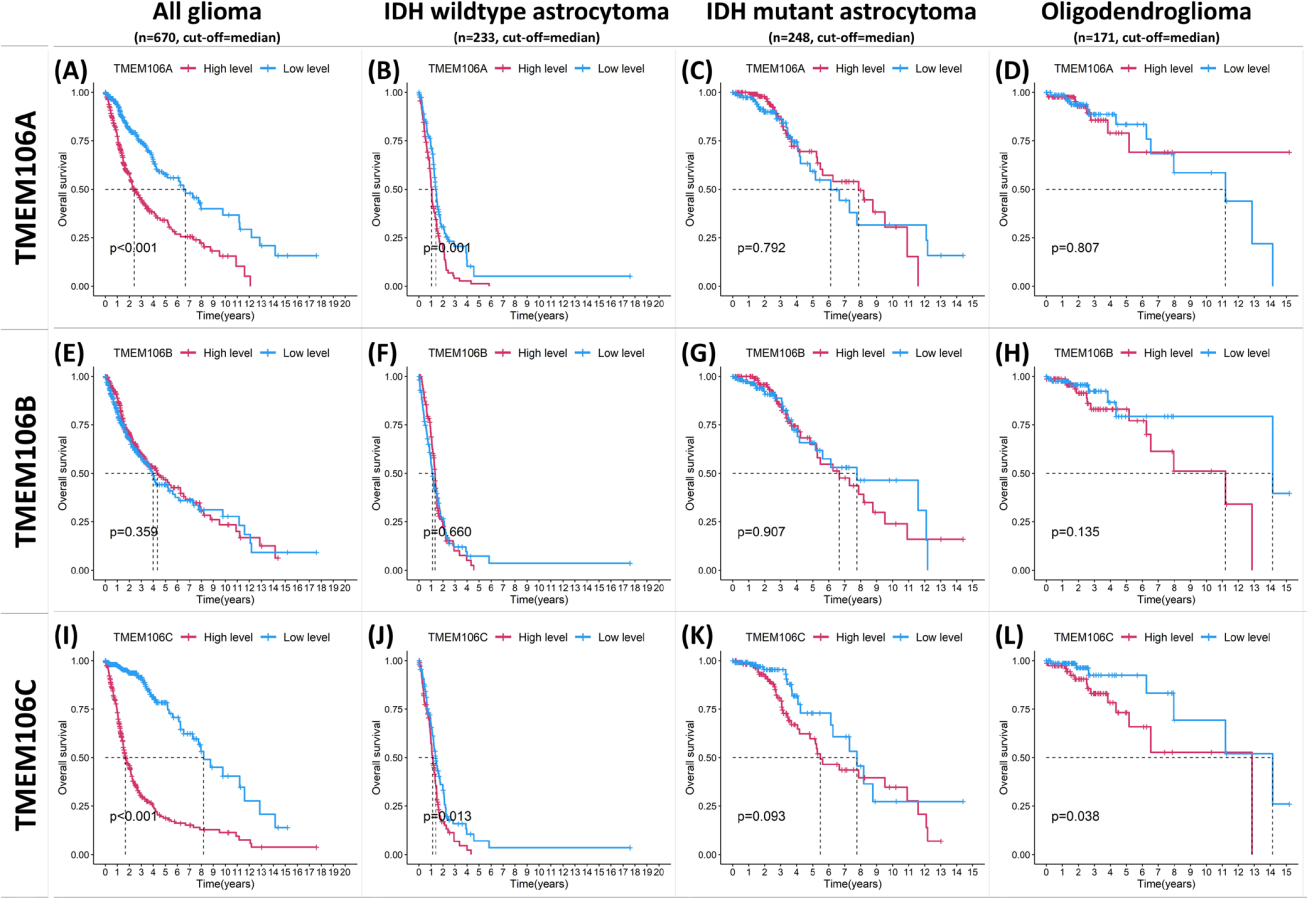
Kaplan–Meier overall survival analyses for (A–D) TMEM106A, (E–H) TMEM106B, and (I–L) TMEM106C. Patients were stratified into high‐ and low‐expression groups based on their respective median levels. Significant survival differences are noted in TMEM106A (A, B) and TMEM106C (I, J, L). *p* < 0.05 indicates statistical significance.

**FIGURE 4:**
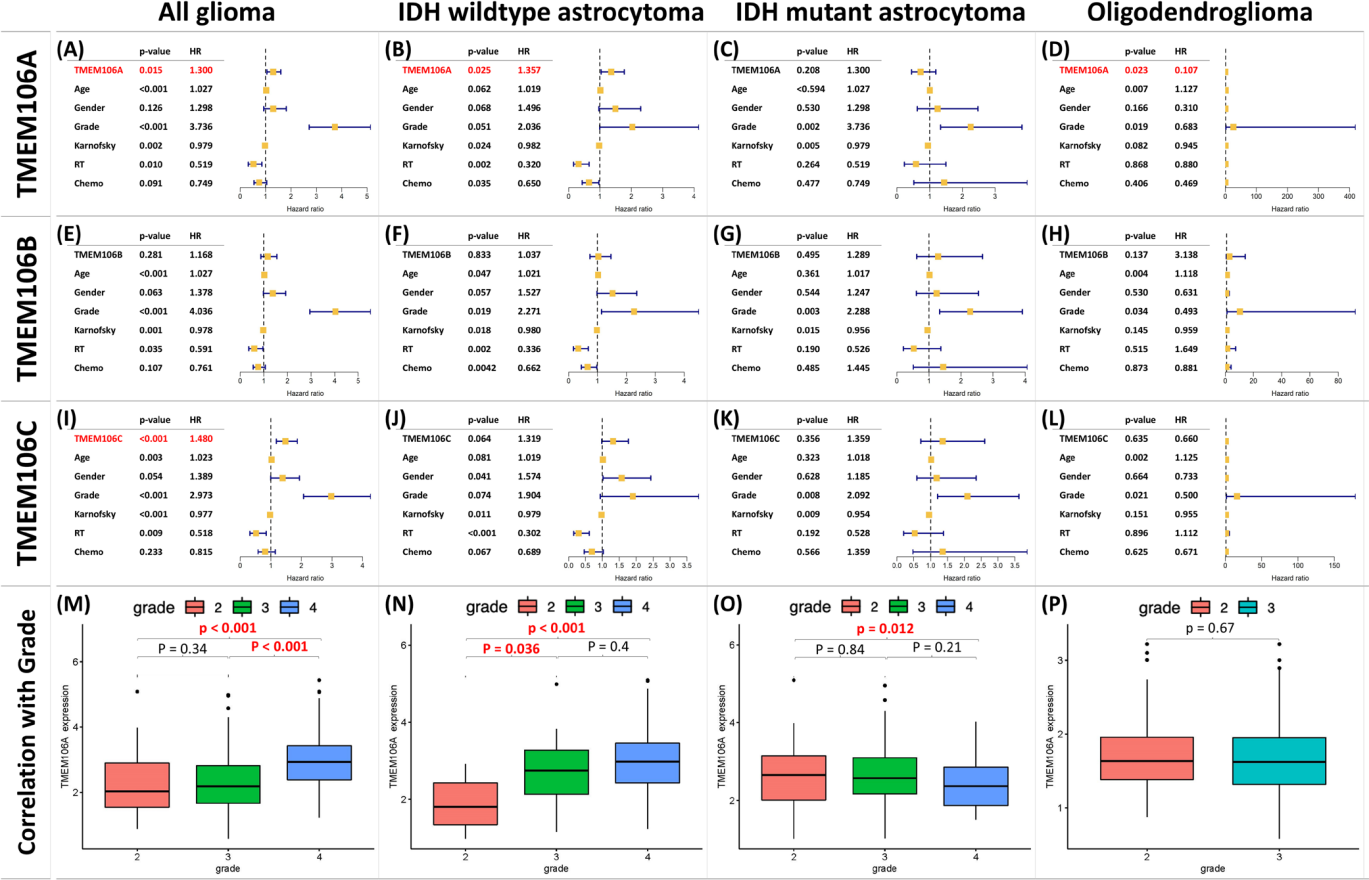
Multivariate Cox regression analyses for (A–D) TMEM106A, (E–H) TMEM106B, and (I–L) TMEM106C, as well as (M–P) correlation between TMEM106A and tumor Grade. *p* < 0.05 indicates statistical significance.

**FIGURE 5:**
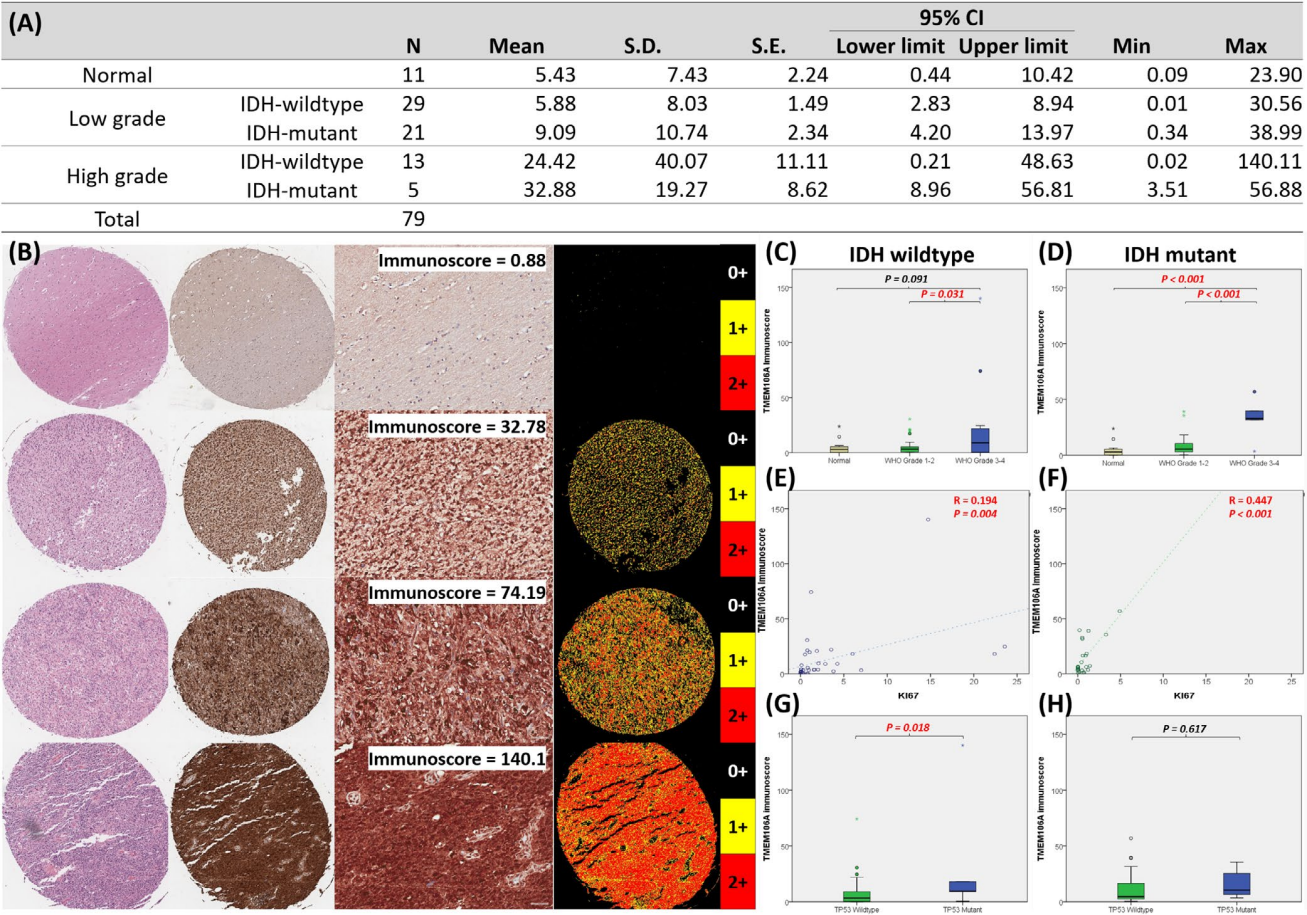
Immunohistochemical quantification of TMEM106A in normal and glioma tissues. (A) Summary of immunoscore for normal (*n* = 11), IDH‐wildtype (*n* = 42), and IDH‐mutant gliomas (*n* = 26), further categorized by WHO grade. (B) Representative TMA cores with varying TMEM106A immunoscore (0.88–140.1). (C, D) Box plots showing TMEM106A levels by WHO grade in IDH‐wildtype or IDH‐mutant tumors. (E, F) Scatter plots reveal a stronger positive correlation between TMEM106A and Ki67 in IDH‐wildtype gliomas. (G, H) Comparison of TMEM106A immunoscore in TP53‐wildtype vs. TP53‐mutant tumors.

**FIGURE 6:**
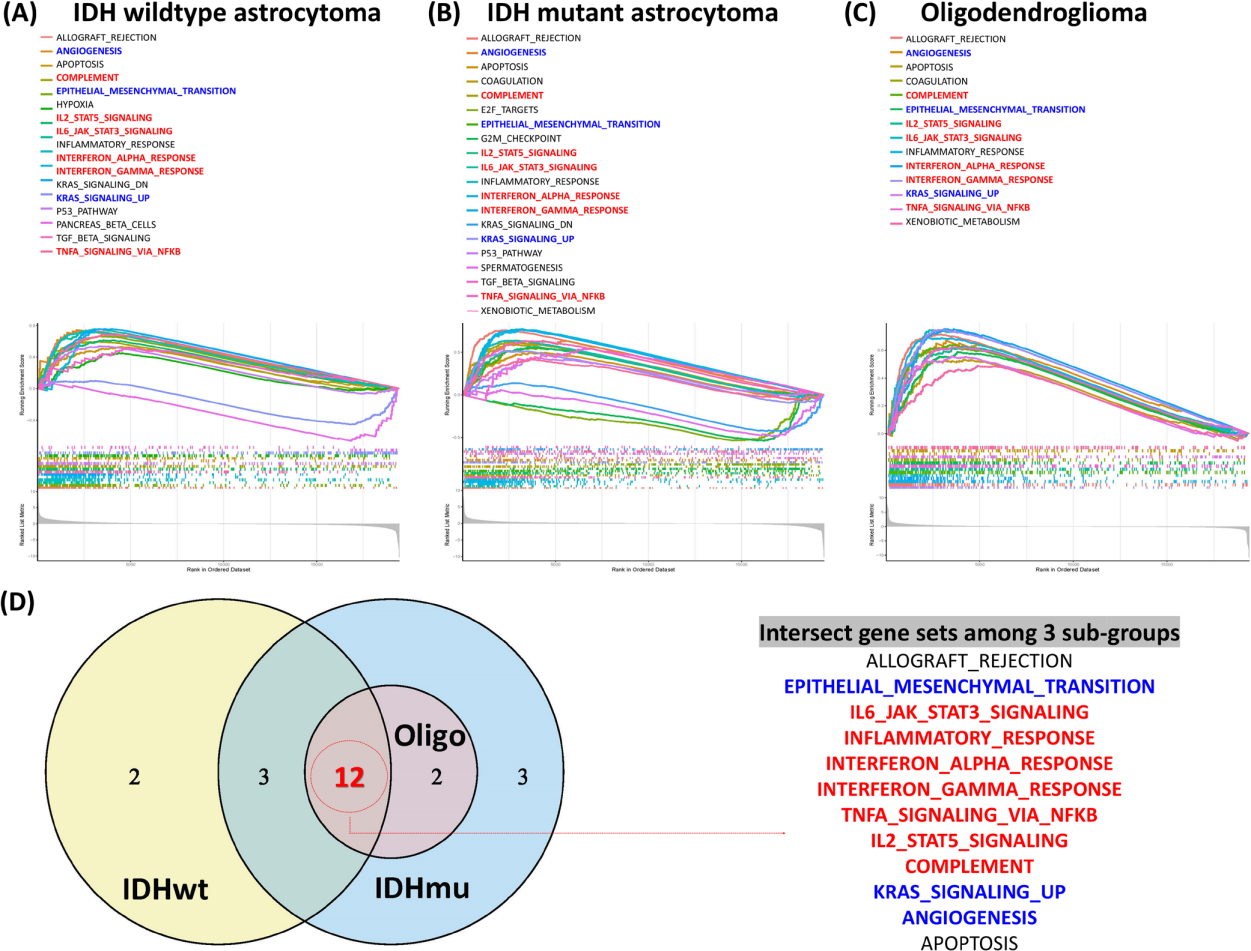
GSEA results for TMEM106A in three glioma subtypes. (A–C) Enrichment plots illustrating hallmark gene sets upregulated in high‐TMEM106A IDH‐wildtype astrocytomas, IDH‐mutant astrocytomas, and oligodendrogliomas. Each curve plots the running enrichment score (*y*‐axis) against the rank‐ordered gene list (*x*‐axis). (D) A Venn diagram illustrates 12 shared pathways among the three subtypes, including multiple immune‐related (red text) and oncogenic signatures (blue text), highlighting a common pro‐inflammatory and pro‐tumor microenvironment associated with elevated TMEM106A expression.

**FIGURE 7:**
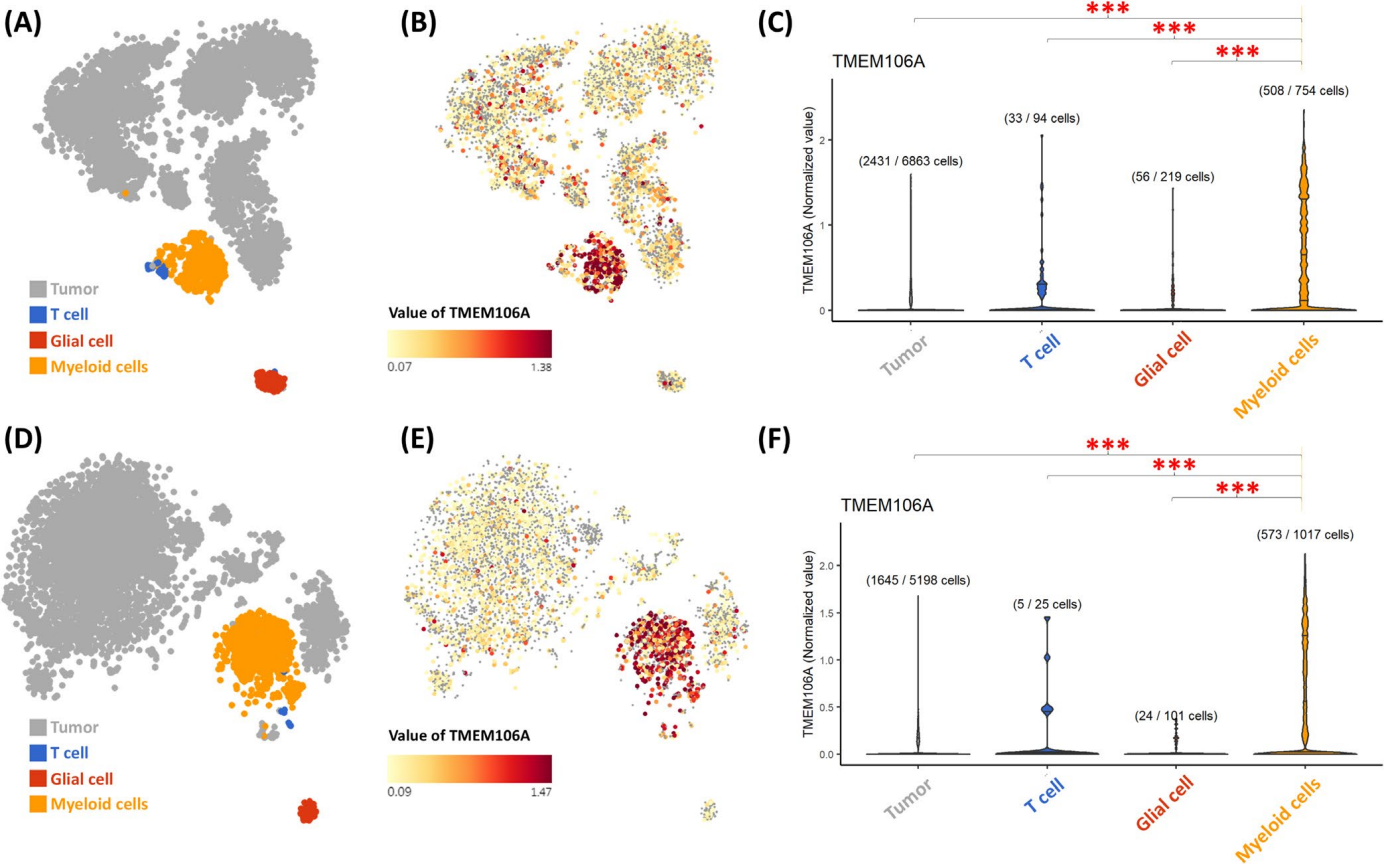
Single‐cell RNA‐seq of TMEM106A in IDH‐wildtype (A–C) and IDH‐mutant (E–G) gliomas. (A, D) UMAP plots color‐code cells by lineage (tumor = gray, T cell = blue, glial = red, myeloid = orange). (B, E) Yellow‐to‐red gradients show the relative magnitude of TMEM106A expression. (C, F) Violin plots reveal significantly higher TMEM106A in myeloid (***p < 0.001).

**FIGURE 8:**
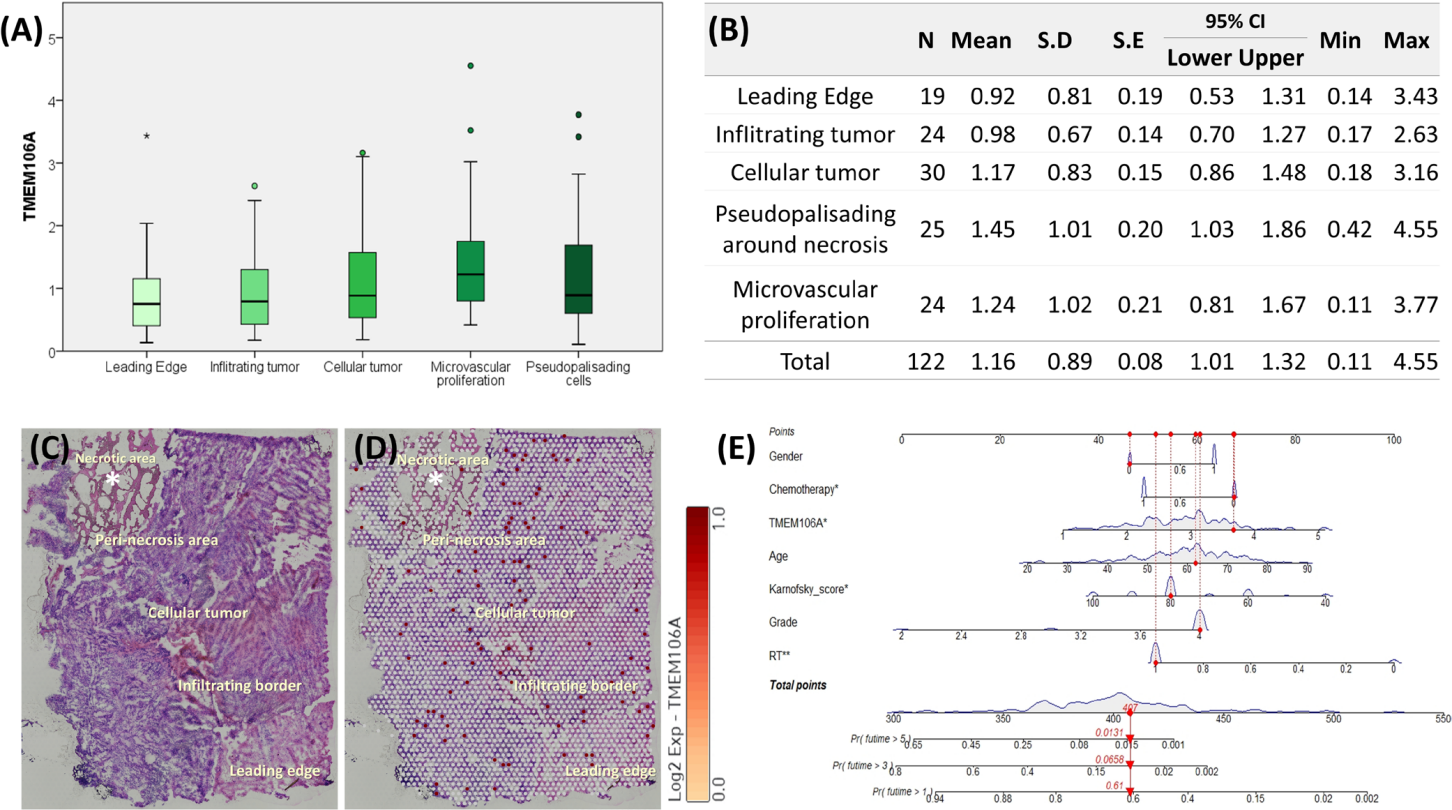
Spatial Distribution of TMEM106A in the Ivy Glioblastoma Atlas. (A) Box plots depicting TMEM106A expression across five histologic subregions: Leading edge (*n* = 19), infiltrating tumor (*n* = 24), cellular tumor (*n* = 30), microvascular proliferation (*n* = 24), and pseudopalisading cells around necrosis (*n* = 25). (B) A summary table displays the mean and confidence intervals for each region, highlighting the progressive increase in TMEM106A from the outer rim to the necrotic center. (C) H&E‐stained section from a human GBM sample featuring multiple tissue cores overlaid with Visium spots. (D) Pseudocolor map of TMEM106A expression at each spot (scale: Yellow = low, red = high). The asterisk indicated the necrotic area. (E) The Nomogram incorporates TMEM106A expression, gender, chemotherapy, age, Karnofsky performance score, tumor grade, and radiotherapy.

**FIGURE 9:**
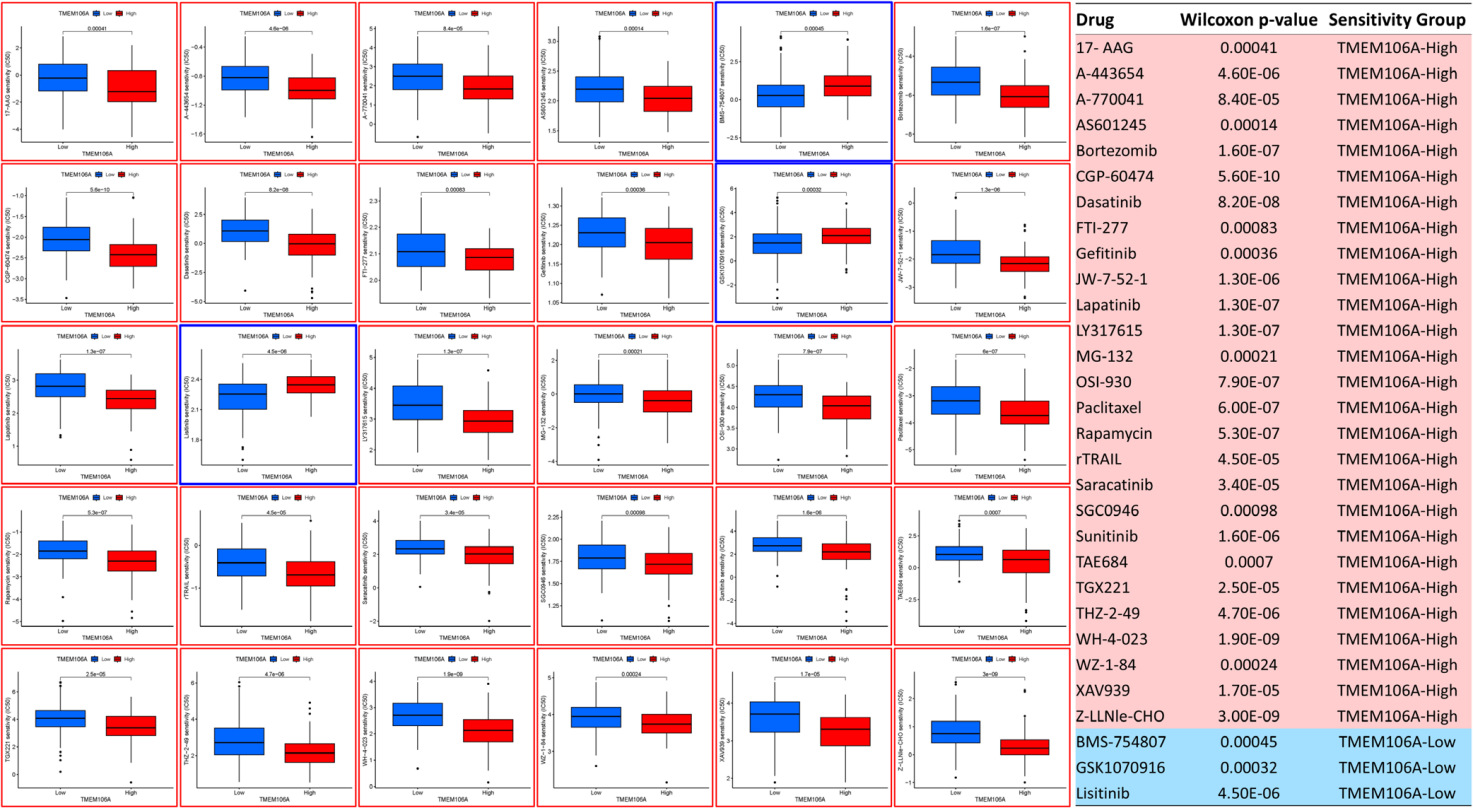
Drug sensitivity inference by pRRophetic. Predicted drug sensitivity (log IC50) for TMEM106A‐Low (blue) versus TMEM106A‐High (red) IDH‐wt tumors using pRRophetic. Samples were median‐split by TMEM106A with two‐sided Wilcoxon *p*‐values (threshold *p* < 0.001). Twenty‐seven agents show lower IC50 in TMEM106A‐High (e.g., dasatinib, lapatinib/gefitinib, bortezomib, rapamycin, and 17‐AAG), while three agents favor TMEM106A‐Low (lisitinib, GSK1070916, and BMS‐754807).

**FIGURE 10:**
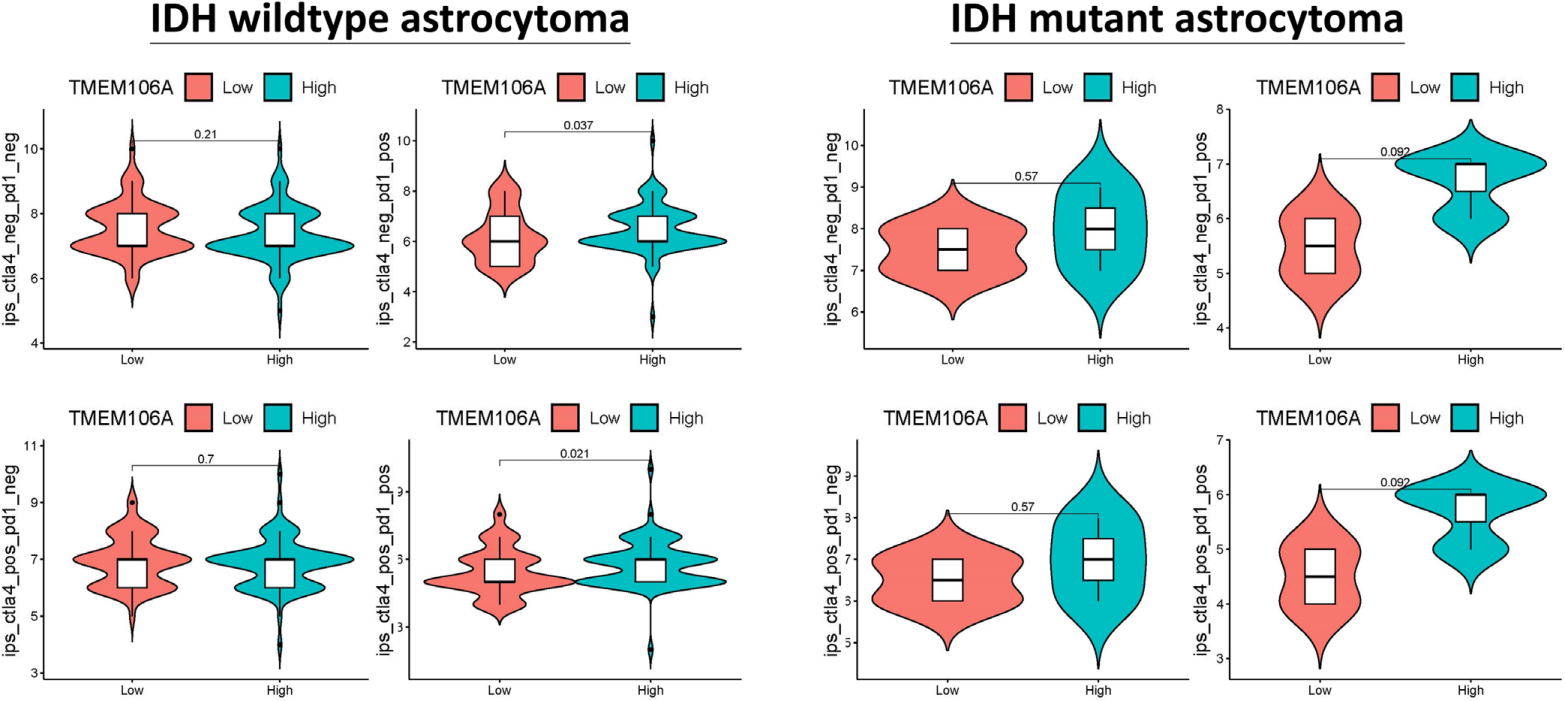
TMEM106A and immunophenoscore (IPS) across checkpoint contexts. Violin plots (with embedded box) compare IPS between TMEM106A‐Low (salmon) and TMEM106A‐High (teal) within IDH‐wildtype (left) and IDH‐mutant (right) astrocytomas across four scenarios: CTLA‐4/PD‐1 off, PD‐1 on only, CTLA‐4 on only, and both on. P‐values are from two‐sided Wilcoxon tests. TMEM106A‐high IDH‐wildtype tumors show significantly higher IPS when PD‐1 is on (*p* = 0.037; *p* = 0.021), while IDH‐mutant tumors show weaker trends.

Figure 1 (TCGA pan‐cancer RNA‐seq) through Timer2.0 platform [16]; Figures 2, 3, 4 (TCGA glioma RNA‐seq); Figure 5 (Biomax GL1001a IHC); Figure 6 (TCGA glioma RNA‐seq with GSEA analyses); Figure 7 (scRNA‐seq: GSE131928 and GSE89567); Figure 8 (Ivy Glioblastoma Atlas and 10x Visium GBM); Figure 9 (TCGA glioma RNA‐seq → pRRophetic CGP2016); Figure 10 (TCGA glioma RNA‐seq integrated with TCIA IPS). Tables 1 and 2 (TCGA and CGGA glioma RNA‐seq).

**TABLE 1:** Correlation between TMEM106A and 12 principal cell compartments in glioma subtypes.

			IDH wildtype astrocytoma		IDH mutant astrocytoma		Oligodendroglioma	
	TMEM106A	Datasets	TCGA	CGGA	TCGA	CGGA	TCGA	CGGA
		Number (*N*)	233	447	247	352	182	214
Tumor	Stemcell_tumor	Correlation	−0.270**	−0.337****	−0.199**	−0.324***	−0.358***	−0.373***
		*p*	< 0.001	< 0.001	0.002	< 0.001	< 0.001	< 0.001
	Prolif_stemcell_tumor	Correlation	−0.076	−0.117*	−0.113	−0.158**	0.266***	−0.105
		*p*	0.249	0.013	0.076	0.003	< 0.001	0.126
	Differentiated_tumor	Correlation	−0.268***	−0.146**	−0.049	0.118*	0.226**	0.248***
		*p*	< 0.001	0.002	0.442	0.027	0.002	< 0.001
Stroma	Oligodendrocyte	Correlation	−0.119	−0.070	−0.039	0.127*	0.096	0.285***
		*p*	0.070	0.140	0.543	0.017	0.197	< 0.001
	Pericyte	Correlation	−0.008	0.107*	−0.185**	0.109*	0.012	0.061
		*p*	0.899	0.023	0.003	0.041	0.872	0.376
	Endothelial	Correlation	−0.131*	−0.278***	−0.153*	−0.159**	−0.055	0.027
		*p*	0.045	< 0.001	0.016	0.003	0.457	0.692
	Fibroblast	Correlation	0.373***	0.362***	0.122	0.201***	0.257***	0.215**
		*p*	< 0.001	< 0.001	0.055	< 0.001	< 0.001	0.002
Immune	Myeloid	Correlation	0.792***	0.702***	0.776***	0.639***	0.782***	0.542***
		*p*	< 0 .001	< 0.001	< 0.001	< 0.001	< 0.001	< 0.001
	Dendritic_cell	Correlation	−0.092	−0.320***	−0.297***	−0.301***	−0.144	−0.213**
		*p*	0.160	< 0.001	< 0.001	< 0.001	0.052	0.002
	T_cell	Correlation	0.129*	0.186***	−0.065	0.171**	−0.242**	0.332***
		*p*	0.049	< 0.001	0.306	0.001	0.001	< 0.001
	B_cell	Correlation	−0.078	0.028	.c	.b	.c	−0.088
		*p*	0.233	0.552				0.199
	Granulocyte	Correlation	0.575***	0.418***	0.399***	0.254***	0.335***	0.181**
		*p*	< 0.001	< 0.001	< 0.001	< 0.001	< 0.001	0.008

*Note:* *p < 0.05, **p < 0.01, ***p < 0.001. Red coloration indicates a positive correlation; blue indicates a negative correlation.

**TABLE 2:** Correlation *of* TMEM106A expression with 22 immune cell types across glioma subtypes.

TMEM106A	*n*	IDH wildtype astrocytoma		IDH mutant astrocytoma		Oligodendroglioma	
		TCGA	CGGA	TCGA	CGGA	TCGA	CGGA
		49	112	86	136	14	24
B_cells_naive	Correlation	−0.014	0.151	−0.091	0.157	−0.459	.c
	*p*	0.926	0.111	0.404	0.068	0.099	
B_cells_memory	Correlation	−0.203	−0.317**	−0.027	−0.325***	0.066	−0.461*
	*p*	0.162	**0.001**	0.806	**< 0.001**	0.822	**0.027**
Plasma_cells	Correlation	−0.193	−0.127	−0.034	−0.166	0.202	0.117
	*p*	0.184	0.184	0.756	0.053	0.489	0.595
T_cells_CD8	Correlation	0.293*	0.078	0.010	0.035	0.016	0.155
	*p*	**0.041**	0.415	0.927	0.688	0.957	0.480
T_cells_CD4_naive	Correlation	.c	−0.002	−0.035	−0.226**	.c	0.051
	*p*		0.985	0.751	**0.008**		0.816
T_cells_CD4_memory_resting	Correlation	−0.139	0.028	−0.053	0.062	0.238	0.192
	*p*	0.341	0.773	0.628	0.474	0.413	0.380
T_cells_CD4_memory_activated	Correlation	0.001	−0.054	0.251*	−0.126	0.204	0.159
	*p*	0.993	0.573	**0.020**	0.145	0.485	0.468
T_cells_follicular_helper	Correlation	0.058	−0.221*	−0.171	−0.273**	−0.507	−0.528**
	*p*	0.690	**0.019**	0.115	**0.001**	0.064	**0.010**
T_cells_regulatory_Tregs	Correlation	−0.197	−0.146	−0.048	0.095	−0.180	0.190
	*p*	0.175	0.126	0.658	0.271	0.538	0.385
T_cells_gamma_delta	Correlation	0.246	−0.165	−0.108	−0.004	−0.035	0.071
	*p*	0.088	0.082	0.321	0.959	0.906	0.747
NK_cells_resting	Correlation	−0.205	−0.166	−0.012	−0.091	−0.070	0.069
	*p*	0.157	0.081	0.916	0.293	0.811	0.753
NK_cells_activated	Correlation	0.085	−0.061	−0.062	−0.182*	−0.140	0.001
	*p*	0.560	0.521	0.569	**0.034**	0.634	0.996
Monocytes	Correlation	0.250	0.095	0.113	0.161	−0.466	−0.046
	*p*	0.083	0.319	0.300	0.061	0.093	0.834
Macrophages_M0	Correlation	−0.373**	−0.216*	−0.028	−0.183*	0.420	−0.110
	*p*	**0.008**	**0.022**	0.801	**0.033**	0.135	0.616
Macrophages_M1	Correlation	0.362*	0.136	0.266*	0.125	0.145	0.029
	*p*	**0.011**	0.153	**0.013**	0.148	0.621	0.897
Macrophages_M2	Correlation	0.298*	0.280**	0.098	0.356***	0.315	0.200
	*p*	**0.037**	**0.003**	0.369	**< 0.001**	0.272	0.360
Dendritic_cells_resting	Correlation	0.200	0.069	−0.036	0.127	0.020	−0.291
	*p*	0.169	0.472	0.743	0.141	0.945	0.177
Dendritic_cells_activated	Correlation	−0.142	−0.059	0.037	−0.146	.c	0.042
	*p*	0.330	0.537	0.735	0.090		0.849
Mast_cells_resting	Correlation	−0.078	−0.060	0.156	−0.054	0.427	0.063
	*p*	0.593	0.530	0.152	0.535	0.127	0.774
Mast_cells_activated	Correlation	0.072	0.037	−0.160	−0.188*	−0.229	−0.064
	*p*	0.622	0.702	0.142	**0.028**	0.431	0.773
Eosinophils	Correlation	−0.016	0.305**	−0.182	0.008	−0.291	−0.017
	*p*	0.913	**0.001**	0.093	0.924	0.313	0.940
Neutrophils	Correlation	0.322*	0.134	−0.038	0.064	0.106	0.053
	*p*	**0.024**	0.160	0.725	0.456	0.718	0.810

*Note:* *p < 0.05, **p < 0.01, ***p < 0.001. Red coloration indicates a positive correlation; blue indicates a negative correlation.

### 2.2 Transcriptome, Proteomics, and Single‐Cell Sequencing Data Processing

All bioinformatics procedures were performed using R (version 4.1.0; www.r‐project.org) and relevant R packages. Original gene‐expression files provided in RPKM (Reads Per Kilobase per Million) were converted to TPM (Transcripts Per Million). The ggpubr and limma packages were used for mRNA regression analyses and to identify differentially expressed genes (adjusted *p* < 0.05). Kaplan–Meier survival analyses and Cox proportional‐hazards models were then applied to assess the prognostic impact of TMEM106A. Tumor–normal contrasts were performed on log2(TPM + 1) values using two‐sided Wilcoxon rank‐sum tests with Benjamini–Hochberg (BH)/FDR control. Single‐cell sequencing data for GSE131928 and GSE89567 were processed and visualized using BioTuring BBrowser [17]. For the single‐cell analysis, we used the authors' curated, analysis‐ready objects for IDH‐wildtype glioma (GSE131928) and IDH‐mutant glioma (GSE89567). Batch correction and normalization followed the pipelines embedded in these objects; we retained those settings unmodified. Cell types (tumor, myeloid, T cells, and reactive glia) were labeled by the authors. Per‐cell TMEM106A (log‐normalized counts as displayed in BBrowser) was compared between lineages using two‐sided Wilcoxon rank‐sum tests, with BH FDR control across lineages (significance at *q* < 0.05). Reproducibility steps: BBrowser → Public datasets → open GSE131928 or GSE89567 → Analysis view → Gene expression → search “TMEM106A” (ENSG00000184988) → Group by “Cell type (author labels)” → export violin‐plot statistics. Scope separation (bulk vs. single‐cell). Tables 1 and 2 report bulk‐level correlations between TMEM106A expression and inferred cell fractions across tumors; no single‐cell data are used in those tables. The single‐cell analysis evaluates per‐cell TMEM106A expression across author‐labeled lineages (tumor, myeloid, T, and glial) within each dataset; it is not a correlation between bulk expression and cell proportion.

### 2.3 Gene Set Enrichment Analysis (GSEA)

Gene Set Enrichment Analysis (GSEA; https://www.gsea‐msigdb.org/gsea/index.jsp, accessed December 2024) [18] was employed to explore pathways differentially altered between high‐ and low‐TMEM106A expression (determined by the median threshold) in the TCGA glioma dataset. The “h.all.v2024.1.Hs.symbols” (accessed December 2024) was selected as the reference gene set, with all additional parameters set to their default values. We used phenotype permutations (*n* = 1000) with the classic scoring scheme and considered gene sets significant at FDR *q* < 0.25 with |NES| > 1.

### 2.4 Twelve‐Cell State and CIBERSORT Analyses

Tumor cell composition was characterized using two deconvolution strategies. The twelve‐cell state method, introduced by Roel G. W. Verhaak [19], detects 12 overarching cell types in bulk tumor samples, whereas CIBERSORT (https://cibersort.stanford.edu/) [20] estimates the relative abundances of 22 immune cell subpopulations from mixed gene‐expression data. Transcriptomic data from TCGA, CGGA, and the Ivy datasets were analyzed to infer the proportions of these 12 cell types and 22 immune cells. For CIBERSORT, we used the LM22 signature with 1000 permutations and quantile normalization disabled for RNA‐seq, and we retained only samples with CIBERSORT deconvolution *p* < 0.05 for downstream cell‐fraction analyses. Associations between TMEM106A and inferred cell fractions were assessed using Spearman's *ρ* with BH correction across the 12‐cell and 22‐cell panels.

### 2.5 Tissue Microarray and Immunohistochemistry

A human glioma tissue microarray (GL1001a) was sourced from Biomax Inc. (https://www.biomax.us/), encompassing 68 glioma specimens (7 Grade 1, 43 Grade 2, 9 Grade 3, and 9 Grade 4) in addition to 11 normal brain tissues. Each core measured 1.5 mm in diameter and was sectioned at a thickness of 5 μm. All samples were collected with informed consent. For consistent staining, a Ventana Benchmark XT immunostainer was used. After deparaffinization, formalin‐fixed sections underwent antigen retrieval via a pressure cooker at 125°C for 30 min (0.01 M sodium citrate, pH 6.2), followed by three 5‐min rinses in PBS. Slides were then placed on the autostainer per the manufacturer's guidelines. Primary antibodies against TMEM106A (Abcam #ab140192), Ki‐67 (Abcam #ab15580), and p53 (Cell Signaling #2524) were used, with detection carried out using the Roche Diagnostics OptiView DAB IHC Detection Kit. The specificity of the antibodies was verified using both positive and negative controls under identical conditions.

### 2.6 Scoring of the Immunohistochemistry

Tissue sections were assessed semi‐quantitatively using previously published methods [7, 8, 9, 21, 22, 23]. Whole‐slide images were captured at 10× magnification, stored as TIFF files, and processed in ImageJ Fiji (https://imagej.net/software/fiji/downloads). Custom macros automatically determined the proportion of tissue displaying three intensity categories (0, 1+, 2+) and calculated the total stained area (0%–100%). The TMEM106A Immunoscore was computed as the area‐weighted staining intensity, that is, the sum of (intensity × percentage of stained area at that intensity) across the three categories (0, 1+, and 2+). Scores range from 0 to 200, and per‐case scores are provided in Data S1. Group differences were assessed by one‐way ANOVA with Bonferroni; when normality or homoscedasticity assumptions (Shapiro–Wilk, Levene) were violated, Kruskal–Wallis with Dunn post hoc and BH correction was used. Correlations with continuous markers (e.g., Ki‐67) used Spearman's *ρ*.

### 2.7 Spatial Transcriptome Data

To visualize TMEM106A expression in situ, a publicly available 10x Genomics Visium dataset for an adult glioblastoma was examined (https://www.10xgenomics.com/datasets/human‐glioblastoma‐whole‐transcriptome‐analysis‐1‐standard‐1‐2‐0). This sample consisted of roughly 3468 spots, each capturing thousands of transcripts [24]. A log2‐transformed TMEM106A expression map was created, illustrating distinct expression distributions near necrotic zones, peri‐necrotic regions, the cellular tumor core, the infiltrating border, and the leading edge. Comparisons of TMEM106A across Ivy subregions used Kruskal–Wallis tests with Dunn–BH post hoc. For 10x Visium, spot‐level contrasts across annotated histologic zones used the same framework with BH correction.

### 2.8 Protein–Protein Interaction Curation and Prioritization

We queried BioGRID [25] for human TMEM106A and restricted to physical interactions from BioPlex affinity‐purification mass spectrometry (AP‐MS) in HEK293T or HCT116. For each record, we extracted the CompPASS score and the cell‐line–specific “top 2%” threshold reported by BioGRID. Interactors were retained only if the score exceeded the threshold for that cell line. These AP‐MS neighbors are used descriptively (hypothesis‐generating) and are not interpreted as glioma‐specific physical binding. (BioGRID; BioPlex AP‐MS). BioGRID build 5.0.250 (released Oct 1, 2025) accessed Oct 19, 2025.

### 2.9 Drug Sensitivity Inference by pRRophetic

Cohort and expression matrix. Bulk RNA seq TPM data from TCGA were used. Gene rows were deduplicated with limma, and genes with low expression were removed (rowMeans > 0.5 in TPM). TCGA barcodes were trimmed to patient level, and tumor samples were retained using the fourth barcode field (primary tumor; normals excluded). For each tumor, TMEM106A expression was extracted, and samples were dichotomized at the median into TMEM106A High and TMEM106A Low. Then, we applied pRRophetic [12] with the CGP2016 training set (cgp2016ExprRma; dataset = “cgp2016”, selection = 1) to predict log(IC50) for each drug contained in drugData2016. In the statistical analysis, we compared the predicted IC50 between TMEM106A groups using a two‐sided Wilcoxon rank‐sum test. Multiple testing was controlled across compounds using BH (FDR < 0.05); we also report raw *P* for transparency (a priori pFilter = 0.001). Extreme predictions were trimmed at the 99th percentile before testing. Directionality was defined as “more sensitive” for the group with a lower predicted IC50—the R code provided in the (Data S2).

### 2.10 Immunotherapy Biomarkers and IPS Modeling

We integrated TCGA expression with TCIA immunophenoscore (IPS) and clinical annotations. Tumor samples were harmonized to patient‐level barcodes. Within IDH‐wildtype and IDH‐mutant strata, tumors were median‐split into TMEM106A‐High/Low. IPS endpoints included the four standard composites: ips_ctla4_neg_pd1_neg, ips_ctla4_neg_pd1_pos, ips_ctla4_pos_pd1_neg, and ips_ctla4_pos_pd1_pos. PD‐L1 was proxied by CD274 log2(TPM + 1); TMB (nonsynonymous/Mb) was retrieved from TCIA, log‐transformed, and *z*‐scored. Within IDH‐wildtype and IDH‐mutant strata, we applied BH correction across the four IPS endpoints (FDR < 0.05); effect sizes are reported as rank‐biserial r for Wilcoxon tests.

### 2.11 Nomogram Construction

For the survival nomogram, we used the TCGA glioma cohort as the modeling dataset. For each tumor, TMEM106A expression was summarized from bulk RNA‐seq as log2 (TPM + 1) and then standardized to a mean‐zero, unit‐variance *z*‐score across the cohort. A multivariable Cox proportional‐hazards model for overall survival was fit with the following covariates: TMEM106A (continuous, *z*‐scored), age at diagnosis (continuous), Karnofsky performance score (continuous), WHO grade (I–IV, modeled as a categorical factor), gender (male/female, factor), radiotherapy (yes/no, factor), and chemotherapy (yes/no, factor). The model was implemented using the rms package in R (function cph) and visualized as a points‐based nomogram with the nomogram() function, similar to previously published glioma nomograms that integrate molecular signatures with clinical variables [26]. In the resulting nomogram, each variable contributes a number of points, and the sum of these points maps to predicted 1‐, 3‐, and 5‐year overall survival probabilities. This model was built at the patient level from bulk TCGA samples; regional expression data from the Ivy Glioblastoma Atlas or 10x Visium were not used. We did not perform formal internal or external validation (e.g., concordance indices and calibration curves), nor did we compare this model against a clinical‐variables–only nomogram; the tool is therefore presented as an exploratory proof‐of‐concept.

## 3 Results

### 3.1 Result 1: Dual Expression Roles of TMEM106A in Cancer: Elevated Expression in Glioma

A comprehensive pan‐cancer analysis of The Cancer Genome Atlas (TCGA) dataset indicates a partial tumor‐suppressive function of TMEM106A, evidenced by significant mRNA downregulation in BLCA, COAD, KICH, KIRP, LUSC, PRAD, READ, THCA, and UCEC (Figure 1A). Conversely, its upregulation in COAD, ESCA, HNSC, LUAD, STAD, and glioblastoma (GBM) suggests TMEM106A's potential role in the aggressive biology of gliomas. For lower‐grade glioma (LGG), although there is a trend toward increased levels compared to normal brain, it lacks statistical significance due to the very limited number of normal brain samples in the TCGA dataset (*n* = 5). A summarized table (Figure 1B) categorizes each cancer type by direction (up‐ or down‐regulation) with a significance symbol; from this, TMEM106A appears to play a potential role in glioma. To comprehensively evaluate TMEM106A's role in glioma, we began by examining all its relatives from the entire TMEM106 family (including TMEM106A, TMEM106B, and TMEM106C) within the context of glioma.

### 3.2 Result 2: Differential Analyses Between Normal and Tumor Tissues Reveal the Essential Roles of *TMEM106A* and *TMEM106C* in Gliomas, Particularly Within the IDH‐Wildtype Subtype

In the differential analyses between normal and tumor tissues, we examined the updated glioma classification, encompassing all gliomas, IDH‐wildtype astrocytomas (IDHwt), IDH‐mutant astrocytomas (IDHmu), and oligodendrogliomas (Oligo), to assess the mRNA expression levels of *TMEM106A, TMEM106B, and TMEM106C*. For *TMEM106A*, the all‐glioma (Figure 2A), IDHwt (Figure 2B), and IDHmu (Figure 2C) groups demonstrated significant differences, whereas the Oligo group (Figure 2D) did not. In the *TMEM106B* study (Figure 2E–H), none of the four groups exhibited significant differences. In the *TMEM106C* analyses (Figure 2I–L), only the IDHwt group (Figure 2J) showed a significant difference, while the remaining groups did not. These findings suggest that *TMEM106A* and *TMEM106C* may play essential roles in glioma, particularly within the IDHwt subtype.

### 3.3 Result 3: High *TMEM106A* and *TMEM106C* Expression Serves as a Prognostic Indicator in Gliomas, Particularly Within the IDH‐Wildtype Subtype

To evaluate the prognostic significance of *TMEM106A*, *TMEM106B*, and *TMEM106C*, Kaplan–Meier survival curves were generated (Data S3), stratifying patients into high‐ and low‐expression groups based on median expression levels. For *TMEM106A*, significant differences were observed in the all‐glioma (Figure 3A) and IDHwt groups (Figure 3B), while the IDHmu (Figure 3C) and Oligo groups (Figure 3D) did not show significant differences. In contrast, no significant differences appeared for *TMEM106B* across any group (Figure 3E–H). Regarding *TMEM106C* (Figure 3I–L), the all‐glioma (Figure 3I), IDHwt (Figure 3J), and Oligo groups (Figure 3L) exhibited significant differences, whereas the IDHmu group (Figure 3K) did not. These findings suggest that *TMEM106A* and *TMEM106C* may play crucial roles in glioma, particularly within the IDHwt, consistent with previous analyses.

### 3.4 Result 4: TMEM106A Emerges as an Independent Prognostic Factor Associated With Aggressive Glioma Behavior, Especially in IDH‐Wildtype Astrocytomas

To account for potential confounding clinical variables, we performed a multivariate Cox regression analysis (Figure 4A–L) that incorporated tumor grade, age, gender, Karnofsky Performance Status (KPS), radiation, and chemotherapy. For *TMEM106A*, significant differences emerged in the all‐glioma (Figure 4A), IDHwt (Figure 4B), and Oligo groups (Figure 4D), while the IDHmu (Figure 4C) did not. In the *TMEM106B* analyses (Figure 4E–H), none of the four groups showed significant differences. For *TMEM106C* (Figure 4I–L), only the all‐glioma group (Figure 4I) demonstrated an important difference, while the other subgroups did not. Collectively, these findings emphasize *TMEM106A* as an independent prognostic marker relative to the other *TMEM106* family genes, including *TMEM106B* and *TMEM106C*. Next, we investigated the relationship between *TMEM106A* and tumor grade (Figure 4M–P). The box plots reveal that *TMEM106A* is associated with tumor grade, particularly in the all‐glioma, IDHwt, and IDHmu groups (Figure 4M–O). Taken together, *TMEM106A* serves as an independent prognostic factor and is associated with more aggressive glioma behavior, underscoring its potential prognostic utility, particularly in IDH‐wildtype astrocytomas (IDHwt).

### 3.5 Result 5: Immunohistochemistry Confirms TMEM106A Upregulation in IDH‐Wildtype Gliomas

To extend our transcriptomic findings from the TCGA and CGGA datasets, we evaluated TMEM106A protein levels using a proteomics approach and immunohistochemical (IHC) staining on clinical samples. A total of 79 cases were analyzed (11 normal, 29 IDH‐wildtype low‐grade, 21 IDH‐mutant low‐grade, 13 IDH‐wildtype high‐grade, and 5 IDH‐mutant high‐grade) to reflect the current molecular classification of gliomas (Figure 5A). Immunohistochemistry scores showed that TMEM106A is most highly expressed in high‐grade tumors, especially in IDH‐wildtype gliomas, whereas the intensity of TMEM106A staining in IDH‐mutant tumors remains comparatively subdued. Representative tissue microarray (TMA) cores are shown (Figure 5B), illustrating the representative TMEM106A immunoscores, from weak (Immunocore = 0.88) to intense (Immunocore = 140.1) staining. Hematoxylin and eosin (H&E) sections provide morphological context, while side‐by‐side immunohistochemical (IHC) images detail the spatial distribution of TMEM106A‐positive cells. Automated quantification further refines this assessment, assigning numerical immunoscores based on the proportion and intensity of brown‐stained tumor cells. To correlate immunostaining with clinicopathological characteristics, we stratified the samples by WHO grade, IDH mutation status (Figure 5C,D), and proliferation index (Figure 5E,F). In both IDH‐wildtype and IDH‐mutant tumors, the TMEM106A immunoscore increases markedly with higher WHO grades and proliferation capability, suggesting an association between TMEM106A expression and malignant progression. We further investigated whether TMEM106A expression interacts with other key glioma markers, specifically TP53 status (Figure 5G,H). Notably, TMEM106A expression showed a significant increase in the TP53 mutation group within the IDH‐wildtype, but no difference was observed in the IDH mutant group. These findings reinforce the notion that TMEM106A may serve as a meaningful biomarker for glioma classification and prognosis, particularly within the IDH‐wildtype subgroup.

### 3.6 Result 6: High TMEM106A Expression Correlates With pro‐Inflammatory and Tumorigenic Signatures

To further elucidate the functional impact of TMEM106A overexpression in gliomas, we performed gene set enrichment analysis (GSEA) across three principal molecular subtypes: IDH‐wildtype astrocytomas (IDHwt), IDH‐mutant astrocytomas (IDHmu), and oligodendrogliomas (Oligo). We first constructed ranked gene lists based on the correlation between the expression level of each gene and the abundance of TMEM106A. By examining the running enrichment scores of hallmark gene sets, we aimed to identify pathways that were significantly activated in tumors with high TMEM106A expression (Figure 6A–C). In IDHwt, 17 hallmark pathways were significantly upregulated in high‐TMEM106A samples, while 20 and 14 pathways showed enrichment in the IDHmu and Oligo groups, respectively. Although the total number of enriched gene sets varied among subtypes, the gene sets themselves revealed considerable overlap. Notably, 12 hallmark pathways were consistently shared among the three glioma subtypes (Figure 6D). These overlapping pathways include several pro‐inflammatory and tumor‐promoting programs, including ALLOGRAFT_REJECTION, EPITHELIAL_MESENCHYMAL_TRANSITION, IL2_STAT5_SIGNALING, IL6_JAK_STAT3_SIGNALING, INFLAMMATORY_RESPONSE, INTERFERON_ALPHA_RESPONSE, INTERFERON_GAMMA_RESPONSE, TNFA_SIGNALING_VIA_NFKB, COMPLEMENT, APOPTOSIS, KRAS_SIGNALING_UP, and ANGIOGENESIS. The prominence of these pathways suggests that high TMEM106A expression is closely tied to a more hostile tumor microenvironment characterized by enhanced inflammation and immune cell infiltration. This environment contains TNFA_SIGNALING_VIA_NFKB, IL2_STAT5_SIGNALING, and IL6_JAK_STAT3_SIGNALING, implying the presence of strong cytokine‐driven pathways. Simultaneously, the identification of EPITHELIAL_MESENCHYMAL_TRANSITION (EMT) and KRAS_SIGNALING_UP highlights TMEM106A‐linked molecular events that are often implicated in tumor invasiveness and metastatic potential. Collectively, these data reveal that TMEM106A‐enriched gliomas exhibit a convergence of pro‐inflammatory and oncogenic signaling pathways, regardless of IDH mutation status. Applying the BioGRID/BioPlex criteria above [27, 28], we highlight four conservative TMEM106A‐proximal proteins (Data S4): ITGA5 (HEK293T; CompPASS 0.929; top‐2% 0.75), ADGRE5/CD97 (HCT116; 0.831; 0.75), DCAKD (HCT116; 0.9997; 0.75), and ADAM21 (HCT116; 0.447; 0.362), which are directionally consistent with our microenvironmental readouts (myeloid enrichment; perivascular/necrosis‐adjacent zones) and our hallmark enrichments (inflammation/interferon/EMT/angiogenesis). Next, we will attempt to elucidate which cellular components are linked to TMEM106A expression and evaluate if targeting TMEM106A‐associated signaling networks could offer clinical benefits.

### 3.7 Result 7: From mRNA TMEM106A‐High Gliomas Show Increased Myeloid and Granulocytes

To elucidate TMEM106A's role in the glioma microenvironment, we performed deconvolution of bulk RNA‐seq datasets to quantify 12 cellular compartments: tumor (tumor‐stem‐like, tumor‐proliferative‐stem‐cell, differentiated‐tumor‐cell), stromal (pericytes, endothelial cells, oligodendrocytes, fibroblasts), and immune (myeloid, dendritic cells, T cells, B cells, granulocytes). In Table 1, each *ρ* (Spearman's rank correlation coefficient) denotes the across‐tumor correlation between bulk TMEM106A expression and the estimated fraction of the indicated compartment in the same tumor (Twelve‐cell state). TMEM106A showed significant positive correlations with myeloid and granulocytic cells in nearly all cohorts, indicating pronounced innate‐immune infiltration; a moderate positive correlation with T‐cell fractions suggests an accompanying adaptive component. Dendritic‐cell fractions generally correlated negatively with TMEM106A. Antigen presentation was not directly evaluated in this analysis. In IDH‐wildtype tumors, TMEM106A correlated with a fibroblast‐like signature in the Twelve‐cell state deconvolution, a signal that in brain datasets commonly represents perivascular/mesenchymal stromal programs rather than canonical fibroblasts. TMEM106A also tended to correlate negatively with populations enriched for stem‐like tumor characteristics, indicating a closer association with inflammatory/reactive microenvironments than with tumor stemness. Overall, high TMEM106A aligned with higher inferred fractions of myeloid and granulocytic cells—and, to a lesser extent, T‐cell and fibroblast‐like signatures—across cohorts, supporting interpretation of bulk TMEM106A as a microenvironmental readout of myeloid‐inflamed niches. Importantly, the Twelve‐cell state and CIBERSORT outputs are compositional: the inferred fractions of tumor, stromal, and immune compartments in each sample are interdependent and must sum to one. As a consequence, higher fractions of myeloid cells necessarily coincide with relative reductions in other compartments (e.g., tumor‐stem‐like or non‐myeloid stromal lineages), and many of the observed correlations between TMEM106A and non‐myeloid compartments are likely mediated, at least in part, by their covariance with the myeloid fraction rather than reflecting fully independent biology. In line with this, we interpret TMEM106A primarily as a readout of myeloid‐inflamed niches, and view its correlations with granulocytic, fibroblast‐like, or T‐cell signatures as secondary features of this broader myeloid‐dominated microenvironment rather than as evidence for distinct compartment‐specific effects.

### 3.8 Result 8: Single‐Cell RNA‐Seq Reveals TMEM106A Enrichment in Myeloid Series

To validate the bulk RNA‐seq and CIBERSORT‐based findings at higher resolution, we examined single‐cell RNA‐seq data from both IDH‐wildtype (Figure 7A–C) and IDH‐mutant (Figure 7D–F) gliomas. Cells were classified into four major lineages—tumor (gray), T cells (blue), glial cells (red), and myeloid cells (orange)—via uniform manifold approximation and projection (UMAP; Figure 7A,D), allowing us to distinguish transcriptionally distinct subsets within the glioma microenvironment. We then visualized TMEM106A expression using color gradients from yellow (low) to red (high) (Figure 7B,E), revealing that numerous myeloid cells showed relatively high TMEM106A levels, followed by T‐cell clusters, whereas the tumor and glial compartments generally exhibited lower expression. Arrows and arrowheads mark areas of particularly strong TMEM106A expression in myeloid and T‐cell populations, respectively (Figure 7A,B,D,E), consistent with TMEM106A mirroring an immune‐active niche. To quantify these differences, violin plots were used to compare TMEM106A levels across all four cell lineages (Figure 7C,F). These plots confirm significantly higher TMEM106A expression (*p* < 0.001) in myeloid cells relative to malignant, glial, and T‐cell populations.

### 3.9 Result 9: TMEM106A Shows Modest Associations With Macrophage Polarization Signatures

Using bulk‐sample correlations across tumors between bulk TMEM106A expression and CIBERSORT‐LM22 inferred immune fractions (Table 2), we observed small‐to‐moderate positive associations with M1 and M2 macrophage signatures across IDH‐wildtype, IDH‐mutant astrocytoma, and oligodendroglioma in TCGA and CGGA (Spearman *ρ* ≈ 0.266–0.362). Conversely, M0 macrophages showed inverse associations (*ρ* ≈ −0.373 to −0.183). Given these modest effect sizes, we interpret the pattern as compatible with higher inferred proportions of polarized macrophages in TMEM106A‐high tumors, without inferring a macrophage‐state shift or causality. Humoral‐immunity components are simultaneously depleted: memory B cells and T follicular helper cells show a significant inverse association in every CGGA dataset and the TCGA oligodendroglioma set. These associations were small in magnitude and cohort‐dependent; we therefore emphasize directional consistency rather than robustness of effect size. These patterns show similar directionality across IDH strata, although magnitudes were modest and some associations were dataset‐specific. In contrast, increases in CD8^+^ or activated CD4^+^ T cells and losses of resting NK, mast, or eosinophil subsets emerge only in isolated datasets, likely reflecting cohort‐specific variability or the limited sample size of oligodendrogliomas (*n* = 14–24). Collectively, these correlative data are consistent with a myeloid‐inflamed microenvironment in TMEM106A‐high tumors and align with the myeloid‐enrichment suggested by previous GSEA results (Figure 6). These findings support TMEM106A as a surrogate marker candidate for myeloid‐rich glioma microenvironments; mechanistic roles remain to be tested.

### 3.10 Result 10: Spatial Stratification of TMEM106A in Glioma: Mapping Expression to Immune Profiles and Clinical Nomograms

To gain deeper insight into the spatial distribution of TMEM106A within the complex architecture of gliomas, we employed two complementary approaches: the publicly available Ivy Glioblastoma Atlas and a high‐resolution 10x Genomics Visium dataset. These techniques enabled us to examine how TMEM106A expression varies across distinct histologic regions, from the relatively less malignant leading edge to the highly proliferative and necrotic tumor core. First, we analyzed data from the Ivy Glioblastoma Atlas, which categorizes glioblastoma tissue samples into five subregions: leading edge, infiltrating tumor, cellular tumor, microvascular proliferation, and pseudopalisading cells surrounding necrosis (Figure 8A,B). Quantitative comparisons revealed that TMEM106A showed a trend of being slightly upregulated in the cellular tumor compartment compared to the leading edge, with further increases observed in microvascular proliferation and only a slight decrease in the pseudopalisading cells surrounding necrosis. These results show that TMEM106A expression is higher in microvascular proliferation and densely cellular tumor regions, compartments that are commonly myeloid‐rich in GBM. To validate and extend these observations, we then used the 10x Genomics Visium Whole Transcriptome platform on a human glioblastoma specimen (Figure 8C,D). Spot‐based transcriptomic profiling allowed us to map TMEM106A expression to thousands of discrete tissue locations. We observed a clear spatial gradient in TMEM106A levels, with higher expression concentrated in peri‐necrotic zones and densely cellular tumor areas, while comparatively lower expression was noted at the tumor edge and necrotic regions. Considering the Twelve‐cell state deconvolution (Table 1), CIBERSORT (Table 2), and single‐cell data (Figure 7A–F) together, TMEM106A shows associations consistent with a myeloid‐enriched microenvironment. We did not compute spot‐level co‐localization with CD68/CD163 transcripts; such spatial benchmarking is prioritized to determine whether TMEM106A marks a distinct or integrated TAM‐rich niche. To illustrate how TMEM106A could be incorporated into risk stratification, we next fit a multivariable Cox proportional‐hazards model in the TCGA glioma cohort with overall survival as the endpoint and covariates TMEM106A expression (per‐tumor log2[TPM + 1] *z*‐score), age, Karnofsky performance score, WHO grade, gender, radiotherapy, and chemotherapy, and visualized this model as a points‐based nomogram (Figure 8E). In this nomogram, each covariate is assigned a point value, and the total points correspond to predicted 1‐, 3‐, and 5‐year survival probabilities for an individual patient [26]. The nomogram is derived from bulk TCGA tumors at the patient level and is not built on Ivy or Visium regional data; TMEM106A is modeled as absolute expression within the cohort and not as expression relative to normal brain. We did not perform formal internal or external validation (e.g., concordance indices and calibration plots) or compare this model against clinical‐only nomograms, so this TMEM106A‐integrated nomogram should be regarded as an exploratory, hypothesis‐generating tool rather than a ready‐to‐use clinical instrument.

### 3.11 Result 11: TMEM106A‐High IDH‐Wt Gliomas Exhibit Broad Predicted Drug Sensitivity

Across TCGA IDH‐wildtype tumors using pRRophetic [12] for drug sensitivity inference, 27 drugs showed lower predicted IC50 in TMEM106A‐high versus TMEM106A‐low (Wilcoxon, two‐sided, *p* < 0.001, Figure 9). Notable classes included SRC family/ABL inhibitors (dasatinib, saracatinib, WH‐4‐023), EGFR/ERBB inhibitors (lapatinib, gefitinib), VEGFR/PDGFR TKIs (sunitinib, OSI‐930), mTOR (rapamycin), proteasome (bortezomib, MG‐132), HSP90 (17‐AAG), CDK (CGP‐60474, THZ‐2‐49), and PI3K/AKT pathway agents (TGX221, A‐443654). rTRAIL also favored TMEM106A‐high. Three drugs were more active in TMEM106A‐low (lisitinib, GSK1070916, BMS‐754807). Predictions used duplicate collapse, low‐expression filtering, 99th‐percentile IC50 trimming, and per‐drug two‐sided Wilcoxon testing (pFilter = 0.001). Because the training corpus comprises cell lines without immune infiltration, these predicted differences should be interpreted as tumor‐cell–intrinsic sensitivity surrogates rather than microenvironment‐aware responses; they are exploratory and require validation in glioma models and, ultimately, clinical cohorts.

### 3.12 Result 12: TMEM106A‐High Associates With Higher Immunophenoscore (IPS) Under PD‐1–Positive Contexts, Especially in IDH‐Wildtype Astrocytoma

Immunophenoscore (IPS) analyses revealed checkpoint‐context–specific enrichment among TMEM106A‐high tumors (Figure 10). In IDH‐wildtype astrocytoma, TMEM106A‐high exhibited significantly higher IPS when PD‐1 was “on”: ips_ctla4_neg_pd1_pos (*p* = 0.037) and ips_ctla4_pos_pd1_pos (*p* = 0.021). In contrast, PD‐1–negative settings showed no difference (ctla4_neg/pd1_neg, *p* = 0.21; ctla4_pos/pd1_neg, *p* = 0.70). These patterns indicate that TMEM106A‐high IDH‐wildtype tumors preferentially align with an immune‐inflamed state likely to benefit from PD‐1–axis blockade. In IDH‐mutant astrocytoma, IPS differences were attenuated: PD‐1–positive comparisons trended higher in TMEM106A‐high (ctla4_neg/pd1_pos and ctla4_pos/pd1_pos, p ≈ 0.09), whereas PD‐1–negative contrasts were null (both *p* = 0.57). Collectively, these data position TMEM106A as a context‐dependent immunotherapy indicator, strongest in IDH‐wildtype disease and PD‐1–on scenarios, consistent with our broader observation that TMEM106A marks a myeloid‐inflamed microenvironment.

## 4 Discussion

### 4.1 TMEM106A as an Independent Prognostic Marker in Glioma: Context‐Dependent Biology Divergent From Other Cancers

Currently, no data addresses the role of the TMEM106 gene family in glioma biology. This gene family contained TMEM106A, TMEM106B, and TMEM106C. From the initial bioinformatics characterization, both TMEM106A and its paralog TMEM106C initially appeared significant in differential expression and survival analyses of glioma. However, only TMEM106A emerged as an independent prognostic marker on multivariate analysis. This suggests that while TMEM106C is upregulated in gliomas and associated with outcomes univariately, its prognostic effect is not independent of other factors. In contrast, TMEM106A retains a unique contribution to patient prognosis. According to previous literature, scholars have identified that TMEM106A loss is enhanced in many cancer types [18, 19, 20, 21] and inhibits tumor cell proliferation and invasion, suggesting a suppressive role. For instance, lung cancer cells exhibit silencing of TMEM106A, and restoring TMEM106A in non‐small cell lung cancer (NSCLC) suppresses proliferation, invasion and induces apoptosis [29]. Similarly, in gastric cancer, TMEM106A is frequently silenced by promoter hypermethylation; forced expression triggers apoptosis and slows tumor growth in vitro and in xenografts [30]. Hepatocellular carcinoma and renal cell cancer also show TMEM106A downregulation linked to tumor progression [31, 32]. A summary of TMEM106A's known mechanisms across cancers is provided in (Table 3) to underscore this dualistic behavior. In glioma, higher bulk TMEM106A expression correlates with higher grade and worse survival, yet single‐cell data indicate the transcript is most abundant in myeloid lineages. The parsimonious interpretation is therefore microenvironmental: bulk TMEM106A levels likely reflect myeloid infiltration rather than a glioma‐cell–intrinsic oncogenic role. We refrain from mechanistic inference. TMEM106A's function might be co‐opted in gliomas to promote tumor aggressiveness, possibly through pathways that are dormant in other tissues of its microenvironment (i.e., immune cells) rather than directly impacting the tumor cells themselves.

**TABLE 3:** Known mechanistic roles of TMEM106A in other cancers.

Cancer type	Experimental Findings (Mechanistic Insights)	Functional	Ref
Non–Small Cell Lung Cancer (NSCLC)	TMEM106A is downregulated in NSCLC. Overexpression reduces proliferation, migration, invasion, and induces apoptosis. TMEM106A also reverses EMT (↑E‐cadherin, ↓N‐cadherin, and vimentin) and inhibits the PI3K–Akt–NFκB pathway.	Tumor suppressor (inhibits growth and EMT)	[29]
Gastric cancer	Silencing via promoter hypermethylation renders TMEM106A absent or low in tumors. Restoring TMEM106A suppresses growth, induces apoptosis, and slows the expansion of xenograft tumors. Re‐expression triggers the activation of caspase‐2, −9, and −3, as well as BID cleavage, leading to PARP inactivation. Approximately 89% of GCs exhibit TMEM106A methylation, which correlates with metastasis.	Tumor suppressor (pro‐apoptotic, epigenetically silenced)	[30]
Renal cell carcinoma (RCC)	TMEM106A is downregulated in RCC. Overexpression slows proliferation and migration while inducing caspase–3–dependent apoptosis. Knockdown in normal cells boosts colony formation. TMEM106A thus acts as a tumor suppressor in RCC.	Tumor suppressor (restricts proliferation & survival)	[31]
Hepatocellular carcinoma (HCC)	TMEM106A is markedly downregulated (often via promoter methylation). Highly metastatic HCC cells exhibit lower TMEM106A levels, and these low levels predict poor postoperative survival. Re‐expression inhibits migration, invasion, and lung metastases, functioning as an anti‐metastatic suppressor.	Tumor suppressor	[32]

### 4.2 TMEM106A as a Macrophage‐Enriched Microenvironmental Readout in Glioma

Across modalities, signals converge on a microenvironmental interpretation of TMEM106A in glioma. In single‐cell datasets, TMEM106A expression is highest in myeloid lineages (Figure 7C,F). Bulk deconvolution links TMEM106A with myeloid and granulocytic compartments (Table 1), and CIBERSORT identifies positive associations with both M1‐ and M2‐polarized macrophage signatures (Table 2). Spatial mapping localizes TMEM106A to peri‐necrotic and microvascular regions (Figure 8), niches that accumulate tumor‐associated macrophages (TAMs) in GBM and align with IL‐6–enriched inflammation [33]. Taken together, these data support viewing bulk TMEM106A primarily as a microenvironmental readout of macrophage‐enriched niches. Relative to canonical markers—CD68 (pan‐macrophage density) and CD163 (M2 skewing)—TMEM106A offers complementary information in two respects: (i) it tracks both M1 and M2 axes (Table 2), indexing the polarization continuum rather than a single pole; and (ii) high TMEM106A co‐occurs with inflammatory and oncogenic programs—IL‐6/JAK/STAT3 and TNF‐α/NF‐κB signaling, EMT, and angiogenesis (Result 6)—and concentrates within the anatomic niches where these programs converge (Figure 8). Collectively, TMEM106A reflects macrophage density, activation state, and spatial context, features that are jointly relevant to outcome in glioma [33, 34, 35]. Head‐to‐head benchmarking with CD68/CD163 and prospective validation will clarify its incremental prognostic and translational utility. Positioning TMEM106A relative to canonical TAM markers. CD68 (pan‐macrophage/microglia) and CD163 (M2‐associated) have been linked to worse outcomes in glioma and are widely used surrogates of TAM burden/polarization [36]. In glioma cohorts, high CD163 likewise predicts poor prognosis [37]. In our study, TMEM106A tracks myeloid enrichment but also co‐aggregates with inflammatory/angiogenic hallmarks and concentrates in peri‐necrotic and microvascular niches—features that suggest a composite niche readout rather than macrophage density alone. To rigorously evaluate incremental value beyond CD68/CD163, a clear next step is to implement nested Cox models (first a baseline with clinical covariates only; next the baseline plus CD68 and CD163; and finally that model plus TMEM106A).

### 4.3 Inflammatory and Oncogenic Programs in TMEM106A‐High Tumors: IL‐6/JAK/STAT3, TNF‐α/NF‐κB, EMT, and Angiogenesis

Beyond cellular composition, TMEM106A‐high tumors exhibited a distinct transcriptional program indicative of heightened inflammation. Gene set enrichment analysis (GSEA) revealed that pro‐inflammatory and cytokine signaling pathways were significantly enriched in TMEM106A‐overexpressing gliomas, most prominently the interleukin‐6/Janus kinase/Signal Transducer and Activator of Transcription 3 (IL‐6/JAK/STAT3) pathway and the Tumor Necrosis Factor‐alpha/Nuclear Factor kappa B (TNF‐α/NF‐κB) pathway. These pathways are well‐known mediators of the immune‐tumor crosstalk in glioma. IL‐6/STAT3 signaling, in particular, plays a central role in glioblastoma progression by driving tumor cell survival and creating an immunosuppressive microenvironment. STAT3 is aberrantly activated in GBM, and IL‐6‐mediated STAT3 activation promotes immune evasion and tumor growth [34]. TNF‐α/NF‐κB signaling is another key inflammatory axis: TNF‐α can stimulate glial and immune cells to produce IL‐6 and activate NF‐κB, which in turn sustains STAT3 signaling, thereby enhancing glioma cell proliferation and aggressiveness [35]. In line with this, immunosuppressive M2‐polarized tumor‐associated macrophages (TAMs) have been shown to paradoxically foster tumor expansion via an NF‐κB/IL‐6/STAT3 positive feedback loop [25], wherein macrophage‐derived cytokines (e.g., IL‐6) activate STAT3 in tumor cells and further skew macrophages toward a tumor‐promoting phenotype. The enrichment of these inflammatory pathways in TMEM106A‐high gliomas suggests that TMEM106A expression is tightly linked to an inflammatory tumor microenvironment, characterized by cytokine activity and macrophage‐driven signaling networks. Applying the BioGRID/BioPlex criteria above [27, 28], we highlight four interactors—CD97, ITGA5/ITGAV axis, ADAM21, and DCAKD—and additionally include SCARB2 from targeted literature (Data S4). These nodes map to adhesion/integrin and pericellular‐remodeling axes, directionally consistent with our microenvironmental readouts (myeloid enrichment; perivascular/necrosis‐adjacent zones) and hallmark enrichments (inflammation/interferon/EMT/angiogenesis). These edges are hypothesis‐generating and are presented as candidates for validation in glioma models.

### 4.4 Translational Outlook: TMEM106A As a Microenvironmental Biomarker for Risk Stratification and PD‐1–Axis Nomination in IDH‐Wildtype Glioma

The identification of TMEM106A as an independent prognostic factor in glioma supports concrete clinical use cases. Viewed as a microenvironmental readout, TMEM106A can aid patient stratification and therapy nomination beyond established classifiers. Consistent with (Figure 10), the “PD‐1–on” specificity of the immunophenoscore (IPS) signal indicates that TMEM106A‐high tumors in IDH‐wildtype astrocytoma align with an inflamed, macrophage‐dominated state that is plausibly compatible with PD‐1–axis benefit. An immediate translational application is TMEM106A‐stratified immunotherapy design. In TMEM106A‐high/IDH‐wildtype disease, PD‐1 blockade emerges as a rational backbone, with biomarker‐guided combinations that address cytokine circuits implicated in our pathway analyses. Given that IL‐6 contributes to glioma immune escape [38], combinations that pair PD‐1/PD‐L1 inhibitors with IL‐6/STAT3 pathway modulators (or related JAK/STAT interventions) merit prospective testing to couple immune reinvigoration with inflammation dampening. The TP53 context offers an additional, testable hypothesis. Our IHC data show higher TMEM106A in TP53‐mutant, IDH‐wildtype tumors (Figure 5G). Contemporary reviews outline strategies to restore wild‐type p53 conformation or destabilize mutant p53 (e.g., APR‐246/eprenetapopt) [39]. Thus, TMEM106A‐high/TP53‐mutant, IDH‐wildtype gliomas represent a rational cohort for mutant‐p53–directed regimens or combinations that integrate PD‐1–axis therapy with p53‐targeted approaches. For risk prediction, incorporating TMEM106A levels (tumor RNA‐seq or IHC) into integrative models can refine clinical decision‐making. Our nomogram (Figure 8E)—combining TMEM106A with gender, chemotherapy, age, Karnofsky performance score, tumor grade, and radiotherapy—illustrates individualized survival estimation and could guide treatment intensity or surveillance frequency. This is particularly useful in IDH‐wildtype lower‐grade gliomas, where TMEM106A‐high status may flag tumors with GBM‐like behavior. Collectively, these observations position TMEM106A as a companion biomarker candidate for risk stratification and PD‐1–axis nomination in IDH‐wildtype glioma, with cytokine‐pathway co‐targeting and mutant‐p53 strategies as prioritized avenues. Prospective studies that standardize assay platforms, define actionable cut‐points, and evaluate predictive value in immunotherapy trials will clarify clinical deployment.

## 5 Limitations

This study is observational and was not designed to demonstrate a glioma‐cell–intrinsic function for TMEM106A. Associations between TMEM106A and inferred immune‐cell fractions were small to moderate (|*ρ*| ≈ 0.18–0.37). These correlations arise from bulk RNA‐seq deconvolution—an indirect estimation approach rather than direct cell measurement—and are not intended to resolve macrophage‐state transitions or causal direction. Head‐to‐head benchmarks against CD68/CD163 for prognosis or PD‐1–contextual IPS were outside the scope of this work. Establishing incremental value and clinically actionable cut‐offs for TMEM106A will require prospective, harmonized cohorts with paired IHC/RNA‐seq and spatial profiling. In addition, the TMEM106A‐integrated nomogram was derived from a single retrospective cohort without formal assessment of discrimination or calibration and without external validation, and it was not benchmarked against clinical‐variable–only models; accordingly, it should be interpreted as hypothesis‐generating rather than as a validated prognostic tool. The candidate interactors were compiled from BioGRID‐curated BioPlex AP‐MS performed in non‐glioma cell lines (HEK293T/HCT116), and one (ADAM21) is from a pre‐publication dataset. Finally, the drug‐response inference uses pRRophetic trained on pan‐cancer cell lines that lack immune and stromal compartments, and we applied it to unadjusted bulk tumor expression. As a result, predicted IC50 differences do not capture macrophage‐driven inflammation, cell–cell interactions, blood–brain barrier constraints, clinical dosing, or toxicity. These outputs should be considered tumor‐cell–centric and hypothesis‐generating; prospective validation in glioma‐relevant experimental systems and patient cohorts is needed. As such, these interactions are hypothesis‐generating and motivate validation in glioma models (e.g., co‐IP/MS, proximity labeling, and spatial co‐localization).

## 6 Conclusion

Across bulk transcriptomics (TCGA/CGGA), single‐cell, spatial, and IHC, we identify TMEM106A as an independent prognostic biomarker in glioma—most pronounced in IDH‐wildtype. Multi‐omic evidence indicates TMEM106A primarily reads out a myeloid‐enriched, inflamed microenvironment, supported by deconvolution, scRNA‐seq, and localization to peri‐necrotic/microvascular niches. GSEA links TMEM106A‐high tumors to IL‐6/JAK/STAT3, TNF‐α/NF‐κB, EMT, and angiogenesis. IPS highlights PD‐1–on enrichment in TMEM106A‐high IDH‐wildtype disease, and pRRophetic predicts sensitivity to several targeted classes. A TMEM106A‐integrated nomogram enables individualized risk. These data support TMEM106A for risk stratification and immunotherapy nomination, warranting prospective validation.

## Author Contributions

**Wen‐Shin Song:** writing – original draft, writing – review and editing, investigation, methodology, funding acquisition. **Pei‐Chi Chang:** methodology, validation. **Dueng‐Yuan Hueng:** funding acquisition, investigation, resources. **Yao‐Feng Li:** writing – original draft, writing – review and editing, methodology, data curation, supervision, project administration, visualization, funding acquisition, investigation, formal analysis, software, conceptualization.

## Funding

This project received funding from the National Science and Technology Council (114‐2314‐B‐016‐020‐MY3 to Y.‐F.L. and 113‐2314‐B‐016‐012‐MY3 to D.‐Y.H.), Tri‐Service General Hospital (TSGH_E_113244 to Y.‐F.L.), and the Ministry of National Defense Medical Affairs Bureau (MND‐MAB‐D‐114136 to Y.‐F.L.). Cheng‐Hsin General Hospital and National Defense Medical University Project (CHNDMC‐114‐04 to W.‐S.S. and Y.‐F.L.).

## Ethics Statement

Ethical clearance was obtained from the Tri‐Service General Hospital Ethics Committee under TSGHIRB No. A202205193 for the project “Brain Tumors Precision Medicine: From Clinical to Genetic and Artificial Intelligence, then Back to Clinical Applications.” This approval encompasses human and animal research, including consent to participate and consent to publish.

## Consent

Written informed consent was obtained from every participant. Immunohistochemistry was performed on the Biomax human glioma microarray (GL1001a) in line with the company's biobanking guidelines.

## Conflicts of Interest

The authors declare no conflicts of interest.

## Supporting information


**Data S1:** Immunohistochemistry quantification of TMEM106A, IDH, P53, KI67 & clinical.

**Data S2:** R code for Drug Sensitivity Inference by pRRophetic.

**Data S3:** Datasets for Kaplan–Meier panel in GBMLGG & IDHwt & IDHmu & Oligo.

**Data S4:** TMEM106A_interactors.

## Data Availability

Large‐scale transcriptomic data were gathered from the GDC portal at TCGA (https://portal.gdc.cancer.gov/) and the CGGA database (http://www.cgga.org.cn/). For spatially resolved tumor analyses, we incorporated the Ivy Glioblastoma Atlas (https://glioblastoma.alleninstitute.org/) and a 10x Genomics Visium Glioblastoma dataset (https://www.10xgenomics.com/datasets/human‐glioblastoma‐whole‐transcriptome‐analysis‐1‐standard‐1‐2‐0). Additionally, single‐cell RNA‐seq data came from the Gene Expression Omnibus (GEO) under accession numbers GSE131928 and GSE89567, enabling more detailed characterization of cell populations within gliomas.
