# Supplementary material for: TMEM106A as a Macrophage‐Associated Biomarker of Prognosis in IDH‐Wildtype Glioma: Integrative Multi‐Omics and Spatial Analyses
Source: Cancer Med. 2025 Dec 7;14(23):e71454. doi: 10.1002/cam4.71454 (PMC12682394; doi:10.1002/cam4.71454)
Supplement: Supplementary file 2 — Data S2: R code for Drug Sensitivity Inference by pRRophetic. [file CAM4-14-e71454-s003.docx]

**~ R code for pRRophetic analyses ~**

## DRUG SENSITIVITY ANALYSIS: TMEM106A-High vs TMEM106A-Low (TCGA IDHwt)

## R >= 4.1; Packages: limma, ggpubr, pRRophetic, ggplot2

## Set your working directory and file names as needed.

suppressPackageStartupMessages({

library(limma)

library(ggpubr)

library(pRRophetic)

library(ggplot2)

})

set.seed(12345)

# ---------------------------

# USER INPUTS

# ---------------------------

expFile <- " your matrix.txt" # your matrix: Gene\tSample1\tSample2...

out_dir <- "drug_sensitivity_outputs" # output folder for figures/tables

gene_of_interest <- "TMEM106A"

p_cutoff <- 0.001 # primary significance threshold

dir.create(out_dir, showWarnings = FALSE, recursive = TRUE)

# ---------------------------

# LOAD CGP TRAINING DATA

# ---------------------------

data(cgp2016ExprRma) # expression models

data(PANCANCER_IC_Tue_Aug_9_15_28_57_2016) # drugData2016 (response metadata)

all_drugs <- unique(drugData2016$Drug.name)

# ---------------------------

# READ AND PREPARE EXPRESSION

# ---------------------------

rt <- read.table(expFile, header = TRUE, sep = "\t", check.names = FALSE, quote = "")

rt <- as.matrix(rt)

rownames(rt) <- rt[, 1]

exp <- rt[, -1, drop = FALSE]

# numeric matrix; average duplicate genes

data_mat <- matrix(as.numeric(as.matrix(exp)), nrow = nrow(exp))

rownames(data_mat) <- rownames(exp)

colnames(data_mat) <- colnames(exp)

data_mat <- avereps(data_mat)

data_mat <- data_mat[rowMeans(data_mat) > 0.5, , drop = FALSE]

# keep tumor samples by TCGA barcode (4th field; first character '0' ~ tumor)

group_code <- sapply(strsplit(colnames(data_mat), "-"), "[", 4)

group_code <- sapply(strsplit(group_code, ""), "[", 1)

group_code <- gsub("2", "1", group_code) # harmonize

data_mat <- data_mat[, group_code == "0", drop = FALSE]

# collapse to patient-level IDs (first three fields)

colnames(data_mat) <- gsub("(.*?)\\-(.*?)\\-(.*?)\\-.*", "\\1-\\2-\\3", colnames(data_mat))

data_mat <- t(avereps(t(data_mat))) # average replicate aliquots per patient

# ---------------------------

# GROUP BY TMEM106A MEDIAN

# ---------------------------

stopifnot(gene_of_interest %in% rownames(data_mat))

tmexp <- as.numeric(data_mat[gene_of_interest, ])

names(tmexp) <- colnames(data_mat)

tm_group <- ifelse(tmexp > median(tmexp, na.rm = TRUE), "High", "Low")

group_df <- data.frame(Sample = names(tmexp), Type = factor(tm_group, levels = c("Low", "High")),

TMEM106A = tmexp, stringsAsFactors = FALSE)

rownames(group_df) <- group_df$Sample

# ---------------------------

# LOOP OVER DRUGS: PREDICT + TEST

# ---------------------------

results <- list()

plot_dir <- file.path(out_dir, "per_drug_boxplots")

dir.create(plot_dir, showWarnings = FALSE)

for (drug in all_drugs) {

# Try to predict log(IC50) using pRRophetic

pred <- tryCatch(

pRRopheticPredict(testMatrix = data_mat, drug = drug, selection = 1, dataset = "cgp2016"),

error = function(e) NA

)

if (all(is.na(pred))) next

# Drop NaN, trim extreme predictions at 99th percentile

pred <- pred[!is.nan(pred)]

if (length(pred) < 10) next

q99 <- stats::quantile(pred, 0.99, na.rm = TRUE)

pred[pred > q99] <- q99

# Align to samples with TMEM106A labels

common <- intersect(names(pred), rownames(group_df))

if (length(common) < 10) next

df <- data.frame(Type = group_df[common, "Type", drop = TRUE],

IC50 = pred[common], check.names = FALSE)

# Two-sided Wilcoxon rank-sum (unpaired)

wt <- wilcox.test(IC50 ~ Type, data = df, exact = FALSE)

pval <- wt$p.value

# Record direction: lower IC50 = "more sensitive"

med_low <- median(df$IC50[df$Type == "Low"])

med_high <- median(df$IC50[df$Type == "High"])

direction <- ifelse(med_high < med_low, "High more sensitive", "Low more sensitive")

results[[drug]] <- data.frame(

Drug = drug,

p_value = pval,

median_IC50_Low = med_low,

median_IC50_High = med_high,

delta_median = med_high - med_low, # negative => High group lower IC50

direction = direction,

n_Low = sum(df$Type == "Low"),

n_High = sum(df$Type == "High"),

stringsAsFactors = FALSE

)

# Plot and save a per-drug boxplot PDF

p <- ggboxplot(df, x = "Type", y = "IC50", fill = "Type",

xlab = gene_of_interest,

ylab = paste0(drug, " predicted sensitivity (log IC50)"),

legend.title = gene_of_interest) +

stat_compare_means(method = "wilcox.test") +

theme(plot.title = element_text(hjust = 0.5))

ggsave(filename = file.path(plot_dir, paste0("drugSensitivity.", gsub("/", "_", drug), ".pdf")),

plot = p, width = 5, height = 4.5, useDingbats = FALSE)

}

# ---------------------------

# SUMMARIZE + THRESHOLD

# ---------------------------

if (length(results)) {

res_df <- do.call(rbind, results)

res_df$FDR_BH <- p.adjust(res_df$p_value, method = "BH")

res_df <- res_df[order(res_df$p_value), ]

# Primary threshold p < 0.001 (matches analysis)

sig_df <- subset(res_df, p_value < p_cutoff)

# Write tables

write.csv(res_df, file = file.path(out_dir, "TMEM106A_drug_sensitivity_all_results.csv"), row.names = FALSE)

write.csv(sig_df, file = file.path(out_dir, "TMEM106A_drug_sensitivity_significant_p_lt_0.001.csv"), row.names = FALSE)

# Convenience split

write.csv(subset(sig_df, direction == "High more sensitive"),

file = file.path(out_dir, "TMEM106A_high_more_sensitive_drugs.csv"), row.names = FALSE)

write.csv(subset(sig_df, direction == "Low more sensitive"),

file = file.path(out_dir, "TMEM106A_low_more_sensitive_drugs.csv"), row.names = FALSE)

}
